# *Staphylococcus epidermidis* isolates from atopic or healthy skin have opposite effect on skin cells: potential implication of the AHR pathway modulation

**DOI:** 10.3389/fimmu.2023.1098160

**Published:** 2023-05-26

**Authors:** Leslie Landemaine, Gregory Da Costa, Elsa Fissier, Carine Francis, Stanislas Morand, Jonathan Verbeke, Marie-Laure Michel, Romain Briandet, Harry Sokol, Audrey Gueniche, Dominique Bernard, Jean-Marc Chatel, Luc Aguilar, Philippe Langella, Cecile Clavaud, Mathias L. Richard

**Affiliations:** ^1^ Université Paris-Saclay, INRAE, AgroParisTech, Micalis Institute, Jouy-en-Josas, France; ^2^ L’Oréal Research and Innovation, Aulnay-sous-Bois, France; ^3^ Paris Center for Microbiome Medicine (PaCeMM), Fédération Hospitalo-Universitaire, Paris, France; ^4^ iMEAN, Toulouse, France; ^5^ Sorbonne Université, INSERM UMRS-938, Centre de Recherche Saint-Antoine, Assistance Publique - Hôpitaux de Paris (AP-HP), Paris, France; ^6^ L’Oréal Research and Innovation, Chevilly-Larue, France

**Keywords:** *S. epidermidis*, aryl hydrocarbon receptor, indoles, inflammation, biofilms, proteases, skin microbiome, skin type

## Abstract

**Introduction:**

S*taphylococcus epidermidis* is a commensal bacterium ubiquitously present on human skin. This species is considered as a key member of the healthy skin microbiota, involved in the defense against pathogens, modulating the immune system, and involved in wound repair. Simultaneously, *S. epidermidis* is the second cause of nosocomial infections and an overgrowth of *S. epidermidis* has been described in skin disorders such as atopic dermatitis. Diverse isolates of *S. epidermidis* co-exist on the skin. Elucidating the genetic and phenotypic specificities of these species in skin health and disease is key to better understand their role in various skin conditions. Additionally, the exact mechanisms by which commensals interact with host cells is partially understood. We hypothesized that *S. epidermidis* isolates identified from different skin origins could play distinct roles on skin differentiation and that these effects could be mediated by the aryl hydrocarbon receptor (AhR) pathway.

**Methods:**

For this purpose, a library of 12 strains originated from healthy skin (non-hyperseborrheic (NH) and hyperseborrheic (H) skin types) and disease skin (atopic (AD) skin type) was characterized at the genomic and phenotypic levels.

**Results and discussion:**

Here we showed that strains from atopic lesional skin alter the epidermis structure of a 3D reconstructed skin model whereas strains from NH healthy skin do not. All strains from NH healthy skin induced AhR/OVOL1 path and produced high quantities of indole metabolites in co-culture with NHEK; especially indole-3-aldehyde (IAld) and indole-3-lactic acid (ILA); while AD strains did not induce AhR/OVOL1 path but its inhibitor STAT6 and produced the lowest levels of indoles as compared to the other strains. As a consequence, strains from AD skin altered the differentiation markers FLG and DSG1. The results presented here, on a library of 12 strains, showed that *S. epidermidis* originated from NH healthy skin and atopic skin have opposite effects on the epidermal cohesion and structure and that these differences could be linked to their capacity to produce metabolites, which in turn could activate AHR pathway. Our results on a specific library of strains provide new insights into how *S. epidermidis* may interact with the skin to promote health or disease.

## 1 Background

S*taphylococcus epidermidis* is a coagulase negative commensal bacterium ubiquitously present on human skin and mucous membranes (1, 2). It is the most commonly cultured bacteria in clinical microbiology laboratories and of almost all body sites of healthy individuals (3, 4). *S. epidermidis* presents opposite roles. Its ability to form biofilms on medical devices when implanted and its antibiotic resistance places *S. epidermidis* as the second cause of nosocomial infections (5, 6). On the skin an overgrowth of *S. epidermidis* has been correlated to the severity of dermatological disorders such as atopic dermatitis (AD) (7), Netherton syndrome (NS) (8) and scalp seborrheic dermatitis/dandruff (9, 10). In particular, the pathogenicity of *S. epidermidis* strains from AD lesional skin has been related to the expression of an exocellular protease EcpA under the control of the regulator Agr, a pathogenic trait shared with its related species *Staphylococcus aureus* (11). Simultaneously, *S. epidermidis* is considered as a key member of the healthy skin microbiota. Recent studies have shown its role in the maintenance of an effective skin barrier *in vitro* (12, 13), in the wound healing (14), protection against the colonization by skin pathogens (15–17) and modulation of the immune system (18–20). Recent developments in the field have provided insights into how *S. epidermidis* strains have benefits for the skin. Specifically, particular commensal strains of *S. epidermidis* can produce the exocellular serine protease (Esp) which inhibits *S. aureus* biofilm formation (19); lantibiotics like epidermin and Pep-S which inhibit the growth of *S. aureus* (20) and 6-N-hydroxyaminopurine (6-HAP) which has an anti-proliferative activity against cancer cells and could protect against skin cancer (21).

While the hypothesis has been proposed of a shift in the behavior of *S. epidermidis* from a commensal to a pathogenic bacterium (21), in last years, a new model showing the diversity of strains has been proposed based on large scale comparative genomics studies comparing healthy and infectious strains (22–24). On the skin, genetically diverse strains of *S. epidermidis* co-exist (22). First report on the genetic difference between *S. epidermidis* isolated from healthy and acne skin identified various functions including biofilm formation and antimicrobials production, suggesting isolates specific effects (25). In the case of AD, *S. epidermidis* isolates have shown phenotypic specificities in terms of antimicrobial and protease activities compared to healthy skin isolates. However, the functional study of *S. epidermidis* isolates from pathological and healthy skin remains limited to particular cases and on a limited number of isolates (11, 20, 25). Moreover, in healthy skin, the exact mechanisms by which commensals *S. epidermidis* interact with host cells and specific receptors remain poorly understood and is likely to be multifaceted.

Two main skin receptors to *S. epidermidis* have been reported to date, with limited information on the molecular effectors involved (26). The immune modulatory molecules such as lipoteichoic acid (LTA), cell wall polysaccharides, peptidoglycan and aldehyde dipeptides constituent or produced by *S. epidermidis* can be sensed by TLRs receptors contributing to the relationship between the bacteria and keratinocyte. For example, a *S. epidermidis* LTA suppresses inflammation by binding to TLR2, limiting tissue damage in mice and promoting wound healing (27). A small molecule of <10 kDa secreted from *S. epidermidis* increases the expression of antimicrobial peptides (in particular β-defensins) through TLR2 signaling (15). *S. epidermidis*-conditioned media could even sensitize human keratinocytes toward *S. aureus* and amplifies the innate immune response against *S. aureus* (28). A second pathway reported to be involved in mediating epidermal response upon *S. epidermidis* stimulation is the aryl hydrocarbon receptor (AHR) (29). *S. epidermidis* could even activate AHR by secreted molecules <2kDa. AHR is a receptor to environmental pollutants including polycyclic aromatic hydrocarbons, tryptophan derivatives such as the photoproduct 6-formylindolo[3,2-b]carbazole (FICZ), the microbial metabolite Indole-3-Aldehyde (IAld); and xenobiotics (30, 31). Whereas AHR has been involved in various skin disorders such as accelerated aging, skin carcinogenesis and inflammation (30–33) AHR is also essential for skin barrier integrity (34) and its activation has been reported to improve the skin barrier in atopic dermatitis (35). Moreover, Uberoi et al. showed that commensal bacteria isolated from human skin can restore skin barrier function via AHR activation in a mouse model (36). Thus, while TLR2 pathway is associated to the skin innate immune response in general, AHR seems to be more related to skin barrier integrity. Considering the diversity of strains on the skin, it raises questions about the effect of *S. epidermidis* strains from different skin origins on this pathway, and how such strains may affect skin barrier components.

Our aim in this study was to determine if the phenotypic characteristics of the *S. epidermidis* strains isolated from skin was correlated to the type of the skin, healthy or atopic. A large phenotypic characterization was conducted to answer this question and showed: (i) that skin origin can be correlated to a specific phenotype of the *S. epidermidis* isolates; (ii) that the AHR pathway might be responsible for part of the differences in behavior observed; and (iii) it also showed that the AHR ligands production from these strains was strongly influenced by the environmental conditions, especially by the interaction with skin cells.

## 2 Methods

### 2.1 Bacterial strains: subjects skin type and sample collection

*S. epidermidis* isolates used in this study were isolated from skin swabs from individuals belonging to two different cohorts. The studies protocols complied with the World Medical Association Declaration of Helsinki, national and EU regulations and L’Oréal Research and Innovation’s procedures based on ICH guidelines for Good Clinical Practice. All volunteers received verbal and written information concerning the study in accordance with the applicable local regulations, guidelines and the current SOP. The volunteer’s written informed consent to participate in the study was obtained prior to any study related procedure being carried out. All data was analyzed anonymously and steps were taken to protect the identities of all participants.

The first cohort (ARC/COPEG/1125; 2013, France) involved 31 healthy women (18 to 45 years old) with different sebum levels on forehead. The non hyperseborrheic (NH) and hyperseborrheic (H) skin status have been determined by measuring the sebum level with Sebumeter (SM810 Courage & Khazaka, Cologne, Germany) by following manufacturer’s instruction on the middle of one half-forehead; sebum levels ranging from 80 - 120 µg/cm² and greater than 150 µg/cm², were assigned to the NH and H status respectively.

Participants were asked to wash their face with the provided neutral soap without anti-bacterial compounds for 3 days (once per day) prior to sampling. Last shampoo and soap were applied 48 and 24 h respectively before sampling. No other products were allowed on the face until sampling was performed.

Participants did not have acute cutaneous disorders, did not receive antibiotics nor had used topical treatments (including anti-acne and anti-seborrhea treatments) or exfoliating products 1 month prior to sampling, or had intense UV-exposure 3 weeks before the study starts.

The sampling for bacterial isolation was conducted as described earlier (37, 38) in a climate-controlled room (22 °C; 60% humidity). The samples were collected by using sterile cotton-tipped swabs pre-moistened with ST solution (0.15 M NaCl with 0.1% Tween 20). Swabs were rubbed firmly on the forehead for 60 sec to cover a surface area of 2 cm². After sampling, each cotton swab was placed into ST solution, vortexed and the suspension were plated on TSA (Tryptic Soy agar) medium to provide the isolates clones.

The second cohort [2012, France (39)] involved patients with moderate atopic dermatitis (AD) (3 to 39 years old). Disease severity was clinically assessed by the dermatologist using the SCORAD (SCORing Atopic Dermatitis) index and clinical signs of erythema, dryness, desquamation at one or more typical lesional (affected) skin areas and a close non-lesional (unaffected) area. Individuals presenting a SCORAD between 25 and 40 were included in the study. Skin microbiome samples were collected using sterile cotton-tipped swabs pre-moistened as described above at the inclusion visit, prior to any treatment, on adult patients. Sampling was conducted in lesional and the closest non-lesional skin sites of 1 cm^2^ by rubbing firmly for 20 sec. over a 1cm^2^ areas as previously reported. Individual bacteria were isolated from the swabs after plating on TSA medium, identified by 16S rRNA gene amplicon sequencing and stored at -80°C.

In the present study, we have included 12 isolates (**Table 1**) and one public strain ATCC 12228. Eight isolates were originated from healthy subjects, with either H (n=4) or NH (n=3); and one additional bacteria isolated from axillary sweat of a healthy subject to reinforce our conclusion with another healthy skin site. Four isolates were originated from 4 different AD patients, which were sampled in either lesional (n=3) or non lesional (n=1) area.

**Table 1:** Bacterial isolates used in this study.

Skin type	Isolate Name	Sampling area		Cohort
**NC**	ATCC 12228	NC	NC	(Winslow and Winslow 1908)
**Hyperseborrheic**	R10C	forehead	Healthy	ARC/COPEG/1125
	11H	forehead	Healthy	ARC/COPEG/1125
	50D	forehead	Healthy	ARC/COPEG/1125
	492 (PH1-28)	forehead	Healthy	ARC/COPEG/1125
**Atopic**	44	buttock	Atopic (lesion)	Flores et al, 2014
	45A5	left leg	Atopic (lesion)	Flores et al., 2014
	45A6	right arm	Atopic (lesion)	Flores et al., 2014
	48	knee	Atopic (non-lesional)	Flores et al., 2014
Non- hyperseborrheic	52B	forehead	Healthy	ARC/COPEG/1125
	1190	forehead	Healthy	ARC/COPEG/1125
	1191	forehead	Healthy	ARC/COPEG/1125
	45	Right arm	Healthy (sweat)	ARC/COPEG/1125

NC, Strain isolated on skin but body site has not been communicated by ATCC.

### 2.2 Bacterial genome sequencing

High molecular weight genomic DNA was extracted from an overnight culture grown in Trytic Soy Broth at 37°C using the Nucleospin tissue kit (Macherey-Nagel). Genomic DNA sequencing was performed on a HiSeq 2500 system (Illumina, 2-kb Nextera XT library, 2x100b paired-end). Short-reads raw data quality was analyzed with FastQC v0.11.8 (40) and reads cleaned with cutadapt v1.18 (41) and Prinseq v0.20.4 (42) in order to remove adapters in 3’ of the sequences the first 15 bases of each read; reads with low quality bases (phred score < 30) at the end of the reads; reads with undetermined nucleotides and sequences whose mean phred score was less than 30. The genomic DNA was also sequenced on a MinION system (Oxford Nanopore Technologies, R9.4.1 flowcell, Rapid Sequencing SQK-RAD004 library, basecalling with Guppy v4.2.2 in 450bps_fast configuration). A hybrid *de novo* reads assembly approach was used as previously described (43). Read remapping rate was verified using Bowtie v2.3.4.3 (44) by calculating the Reads Mapped Back to Contigs (RMBC) index. Each assembly was annotated using PROKKA v1.13.3 (45). The annotated sequences and raw reads were deposited in the NCBI and SRA databases under the accession numbers listed in **Supplementary Table T1**.

### 2.3 Comparative genome analysis

The core genome was generated following the method described by Christensen et al. (23). Briefly, the ATCC 12228 reference *S. epidermidis* genome was split into 200b non-overlapping fragments and used as Blast query on the 11 *S. epidermidis* genomes sequenced in this study. Each common hit was kept and aligned with MUSCLE v3.8.31 (46) in order to obtain gapped multiple alignments. All the gapped alignments were then concatenated to generate each isolate core genome. Another comparative method used the FastANI v1.31 (47) by calculating the Average Nucleotide Index for each genome pair. Either the core genomes alignment or the ANI distance matrix were used to generate phylogenic trees with FastTree v2.1.11 (48). Phage sequences were identified from the genomes using PHASTER (49). Accumulation-associated protein (*aap*) sequence search was conducted using a Blast query with the 492 isolate *aap* gene (REF). Antimicrobial resistance (ABR) genes were identified using Abricate v1.0.1 tool (50). Default parameters were used for all software unless otherwise specified.

Genome sequences from 811 *S. epidermidis* strains were available from Larson et al. (51). Genomes from each strain described in this study have been sequenced and phylogenic trees were generated using PhyloMEAN by iMEAN, based on phylonium (52) and PHYLIP (53) with default parameters. Isolates described in this study were assigned into the primarily described clades (51) based on their primary branch from an unrooted tree. The cladogram was visualized with Dendroscope (54, 55). Blast analyses were performed on each genome to identify presence of genes implicated in indole metabolites biosynthesis pathway (see **Supplementary Table T7**).

### 2.4 Biofilms analysis

Biofilm analysis was conducted as described earlier (56, 57). Briefly, bacteria were pre-cultured in TSB 3% w/v overnight at 37°C, 170 RPM. 200 µL of each bacteria was deposited at OD=1 (optical density) in duplicate on 96 well plates of black polystyrene with transparent bottom (µClear Greiner Bio-one, France) according to Christensen et al. (58), incubated 1 hour at 37°C aerobically. Then medium was refreshed in order to remove non-adherent microorganisms, and plates were further incubated for 3 days at 37°C, the medium was refreshed every day. Negative controls, comprising wells containing 200 µL of TSB without microbial suspension, were also prepared under the same conditions. At the end of the incubation period, the microorganisms were stained by SYTO™9 (Invitrogen) (diluted 1:350 in TSB from a stock solution at 5 mM in DMSO), a green fluorescent nucleic acid stain that is permeant to bacteria cell membrane, for 45 min at room temperature in the dark. Biofilms were defined as the presence of adherent and cohesive bacteria in the plate after 5 washes. Stained biofilms were examined at the MIMA2 imaging platform with a confocal laser microscope (Leica model HCS SP8; Leica Microsystems CMS GmbH, Mannheim, Germany) using a 40× objective (HC PL FLUOTAR). In order to minimize the air contact and maintain constant sample moisture condition, a coverslip was used on the specimen. A 488 nm argon laser was used to excite SYTO™9, and the emitted green fluorescence was collected in the range 500 to 540 nm using a hybrid detector. 512x512 pixels images from four randomly selected areas of raw biofilms, and two after five manual successive washes with sterile water, were acquired for each well. For each of them, sequential optical sections of 1 µm were collected along the z axis over the complete thickness of the sample to be subsequently analyzed. Biofilm biovolume (in µm^3^) was extracted from image series using the Fiji software (Fiji, ImageJ, Wayne Rasband National Institutes of Health) and rendered into 3D mode using Imaris software (Bitplane AG).

### 2.5 Proteases activity assay

The protease activity assays were performed by using the EnzChek Elastase Assay Kit and EnzChek Gelatinase/Collagenase Assay Kit (Thermo Fisher Scientific) according to the manufacturer’s instructions. Briefly, 10 µL of each bacterial supernatant (12 *S. epidermidis* (**Table 1**) and ATCC 12228) cultivated in 2 mL medium for 24h at 37°C, were incubated with 1 µg of DQelastin or DQgelatin (Thermo Fisher Scientific) diluted in the supplied reaction buffer (qsp 200 µL) in 96-well black plates (Corning, NY) for 20 hours. Relative fluorescent intensity was analyzed with a Fluorometer Infinite^®^ M200 PRO (Tecan system; excitation wavelength, 485 nm; emission wavelength, 538 nm).

### 2.6 Keratinocytes culture and treatments

Normal neonatal human primary epidermal keratinocytes (NHEKs) (CellnTec) were cultured in CnT-57 supplemented medium (CellnTec) containing bovine pituitary extract (BPE) at 37°C and 5% CO_2_. NHEKs were used only for experiments between passages 3 to 5. NHEKs were seeded at 10,000 cells/well in 12 wells plates and then grown to about 80% confluency in keratinocyte-SFM (Gibco, Life Tech. Corp., Grand Island, USA) supplemented with EGF 0,035 µg/µL and BPE 12,4 mg/mL (Gibco) 24 hours before treatments. For bacterial supernatant treatments, NHEKs were treated for 14h with sterile-filtered bacterial supernatant at 50% (vol) (prepared in KSFM medium for 14 hours) and then harvested for RNA extraction. For the treatment with live bacteria, *S. epidermidis* were pre-cultured in keratinocytes culture medium (KSFM) overnight until exponential phase. Bacterial suspensions concentrations were then adjusted to 10^8 CFU/mL (corresponding to Optical Density = 0.18) by adding KSFM and added to the NHEKs at a MOI = 10:1. After 14h of this co-culture, at 37°C and 5% CO_2_ conditioned media (supernatants) were collected and analyzed for IL-8 quantification (IL-8 DuoSet ELISA, R&D systems), cytotoxicity assay (CyQuant™ LDH cytotoxicity assay, Thermo Fisher) following manufacturer’s instruction, while NHEK cells were harvested for RNA extraction for quantitative real-time PCR (qRT-PCR) assays.

### 2.7 3D skin treatments

The human skin equivalent model (HSE) LabSkin (Innovenn, York, UK) (full thickness human skin equivalent incorporating fully-differentiated dermal and epidermal components), are received at day 13 after air-liquid interface exposure and placed in deep wells plates with the medium provided by the manufacturer, overnight at 37°C, 5% CO_2_. HSEs were then treated with 20 µl of a suspension containing 10^3^ UFC of a single *S. epidermidis* isolate [*S. epidermidis* ATCC 12228, or clinical isolates from NH (BC1191, 52B), H (50D) or AD skin (45A6, 44)]. Each strain was pre-cultured in TSB 3% and then resuspended in LabSkin medium before topical application using a sterile loop as described previously (59). Application of 20 µL sterile Labskin medium was inoculated as control. HSEs were then incubated at 37°C, 5% CO_2_ for 2, 5 and 7 days. Medium was refreshed every 2 days. At day 5 and 7, skin conditioned media were collected and submitted to IL-8 quantification, and HSEs were cut in half for quantitative real-time PCR assay and histology analysis (hematoxylin eosin safran HES staining).

### 2.8 Quantitative RT-qPCR

Total RNA was extracted from keratinocytes by using the RNeasy Micro kit (Qiagen, Hilden, Germany) according to the manufacturer’s protocol with an additional DNAse treatment (Rnase-Free Dnase Set, Qiagen). RNA concentration was determined using a Nanodrop™ (Nanodrop 1000, Thermo Scientific). iScript cDNA Synthesis (Biorad) was used to synthesize cDNA. mRNA levels were measured with the StepOnePlus™ Real-Time PCR System (Applied Biosystems™) and SYBR Green Master Mix (Biorad). Quantitative PCR (qPCR) data were analyzed using the 2^−ΔΔ^ *^C^* ^t^ quantification method with GAPDH (glyceraldehyde-3-phosphate deshydrogenase) for eukaryotes cells and GyrB (DNA gyrase subunit B) for *Staphylococcus* as the endogenous control. Primers for FLG, IVL, KLK7, DSG1, CDSN, Ki67, DEFB4, STAT6, OVOL1, OVOL2 were provided by Biorad, the other primers sequences are shown in **Table 2**.

**Table 2:** qPCR primers used in this study.

Gene target	Forward primer 5’-3’	Reverse primer 5’-3’	Reference
CYP1A1	CAG CTC AGC TCA GTA CCT CA	CTT GAG GCC CTG ATT ACC CA	This study
EcpA *Se*	TGT GCT TAA AAC GCC ACG TA	GTA TAG CCG GCA CAC CAA CT	Cau, 2020 (11)
GyrB *Se*	GTT GTA ATT GAG AAA GAC AAT TG	TAC AGT TAA GAT AAC TTC GAC AG	This study

### 2.9 Metabolites quantification

Internal standards were purchased from Santa Cruz Biotechnology (5-Hydroxyindole acetic acid-d5, Serotonin-d4, Indole-3-acetic acid-d4, Kynurenic acid-d5, Melatonin-d4, Piconilic acid-d3, Tryptamine-d4, and Xanthurenic acid-d4) and Toronto Research Chemicals (3-Hydroxyanthranilic acid-d4, 3-Hydroxykynurenine-13C2–15N, 5-Hydroxytryptophan-d4, Indole-3-acetamide-d5, Kynurenine-d4, and Tryptophan-d5). Stock solutions of labeled analytes were prepared in water with 0.1% of formic acid and final concentrations have been chosen to match estimated endogenous metabolites concentrations. Metabolites were extracted from 50 μL of bacteria supernatant or co-culture medium. After addition of the internal standards (100 μL) and 300 μL of MeOH, the samples were vortexed for 15 s and homogenized at -20°C, for 30 min. After centrifugation at 5 000 rpm 10 min at -4°C, 350 μL of supernatant were collected and concentrated under N2 flux. The residues were reconstituted in 100 μL of MeOH/H2O (1:9) and transferred to a 96-well plate for LC-HRMS analysis. Analyses were performed as described previously (60) with some adaptations. Briefly, 2 μL were injected into the LC-MS (XEVO-TQ-XS, Waters^®^). A Kinetex C18 xb column (1.7 μm x 150 mmx 2.1 mm, temperature 55°C) associated with a gradient of two mobile phases (Phase A: Water + 0.5% formic acid; Phase B: MeOH + 0.5% formic acid) at a flow rate of 0.4 mL/min were used. The solvent gradient, the parameters of the Unispray^®^ ion source along with the parameters for fragmentation and analysis of the metabolites and internal standards are given in supplementary data (**Supplementary Table T8** and **Supplementary File_F1**). For each metabolite, a calibration curve was created by calculating the intensity ratio obtained between the metabolite and its internal standard. These calibration curves were then used to determine the concentrations of each metabolite in samples (61).

### 2.10 Statistical analysis

All experiments were performed with at least three biological replicates. GraphPad Prism version 7 (San Diego, CA, USA) was used for all analyses and preparation of graphs. For all data displayed in graphs, the results are expressed as the mean ± SEM. The D’Agostino and Pearson test of normality was applied to all data sets, and in cases where the data did not demonstrate a normal distribution, non-parametric tests were used to analyze statistical differences. For comparisons between two groups, Student’s t test for unpaired data or non-parametric Mann–Whitney test was used. For comparisons between more than two groups, one-way analysis of variance (ANOVA) and *post hoc* Tukey test or nonparametric Kruskal–Wallis test followed by a *post hoc* Dunn’s test was used. For all statistical tests, differences with a p value less than 0.05 were considered statistically significant: *p <0.05, **p <0.01, ***p<0.005, ****p<0.001.

## 3 Results

### 3.1 Genome sequence comparisons show cluster of strains isolated from atopic skin

Twelve *S. epidermidis* were isolated from different subjects depending on the skin type and the disease status (**Table 1**). As it is reported that *S. epidermidis* subtypes differ significantly between skin microenvironment (moist, sebaceous, dry), we selected healthy skin isolates from two skin types: NH and H (1). Three groups were created: healthy skin comprising NH and H skin isolates and AD skin isolates. For comparison the reference strain ATCC 12228, not associated with any type of skin, was used as control strain. The genome sequencing of 11 isolates was completed with Illumina and Nanopore technology, a long read sequencing to improve the genome quality. The sequences were assembled using SPAdes algorithm and automatically annotated using Prokka algorithm. Genome sizes were oscillating between 2,300,583 bp and 2,598,183 bp with a GC content between 31.97% and 32.05% (**Supplementary Table T1**). Two global genomic comparisons were carried out using these assembled genomes, one based on the core genomes alignment as described by Christensen et al. (25), the other based on whole genome sequences using the FastANI algorithm. Using both methods we confirmed that all isolates were *S. epidermidis*.

**Figure 1:**
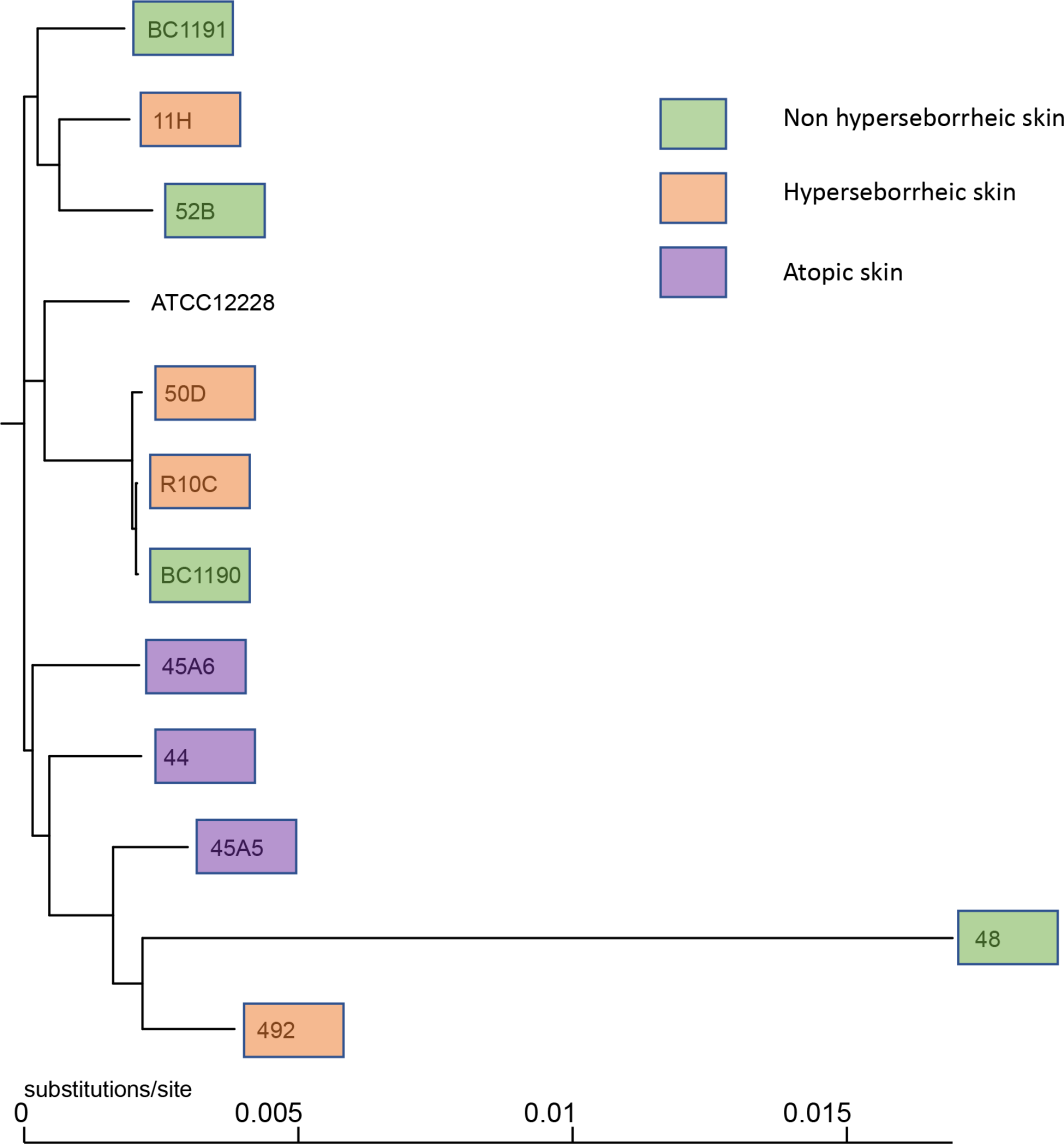
Phylogenetic tree of the 11 core genomes of *S. epidermidis* using strain core genome. The tree was generated using FastTree v2.1.11 (43).

Observation of the phylogenic trees obtained by both methods revealed that the S. epidermidis bacteria isolated from atopic skin were clustering genetically, while others from the two others types of skin were not (**Figure 1** and **Supplementary Figure S1**). Interestingly, the isolate 48 isolated from a non-lesional skin of a subject with atopic dermatitis was genetically different from the AD cluster, using both methods. These data suggest that even if all isolates are *S. epidermidis*, those from atopic skin might have genetic differences.

We then inspected characteristics of *S. epidermidis* genomes associated with their genetic diversity on skin and differentially observed on body sites (24). PHASTER analysis revealed the presence of intact StB20-like prophage in 7 genomes associated to the 3 skin types (NH n= 2; H n=3; AD n= 2) and StB12 prophage in 1 genome associated to AD lesion (**Supplementary Table T2**). Comparison of the 7 StB20-like phage sequences did not show major differences among their genomic structure (**Supplementary Figure S2A**). Search for antimicrobial resistance genes using Abricate v1.0.1 tool, showed differences in the gene patterns (**Supplementary Table T3**), for example isolates 45A5 (AD lesional), 48 (AD non lesional), 492 (H) and 52B (NH) did not carry the *blaZ* beta-lactamase gene present in the reference strain ATCC 12228 strain. However, the differences in antimicrobial resistance (*abr*) genes content did not match with the skin type. Finally, the presence of the accumulation-associated protein (Aap) CDS, coding for a megaprotein protein involved in skin corneocytes adhesion (62) and biofilm formation in commensal *S. epidermidis* strains (63), has been investigated. Additionally, a particular organization of this gene has been reported, regarding the absence of C-terminal collagen triple helix domain (43). Here we confirmed the presence of an Aap CDS in the majority of the genomes (9/11), and 8/9 aap CDS contained- a C-terminal collagen triple helix domain, independently of the skin type of origin (**Supplementary Figure S2B**). This first analysis suggests that genetic specificities of the strains from atopic skin are not related to the presence of prophage, *abr* genes or *aap* CDS.

### 3.2 *S. epidermidis* isolated from patients with atopic skin lesions form smaller biofilms

Biofilm formation is a unique feature developed by microorganisms that permits colonizing various types of biotic or abiotic niches and resists environmental factors allowing the persistence of microorganisms. Biofilm formation of *S. epidermidis* has been studied for many years due to various nosocomial infections caused by biofilm formation on indwelling devices (64, 65) even if not all isolates of *S. epidermidis* form a biofilm (66). There are controversial data concerning the capacity of the model strain *S. epidermidis* ATCC 12228 to form or not biofilms, since originally this strain was described as incapable of forming a biofilm (67). However, it seems from the literature that it depends on the growth conditions as several publications reported a biofilm formed by this strain (68–70).

**Figure 2:**
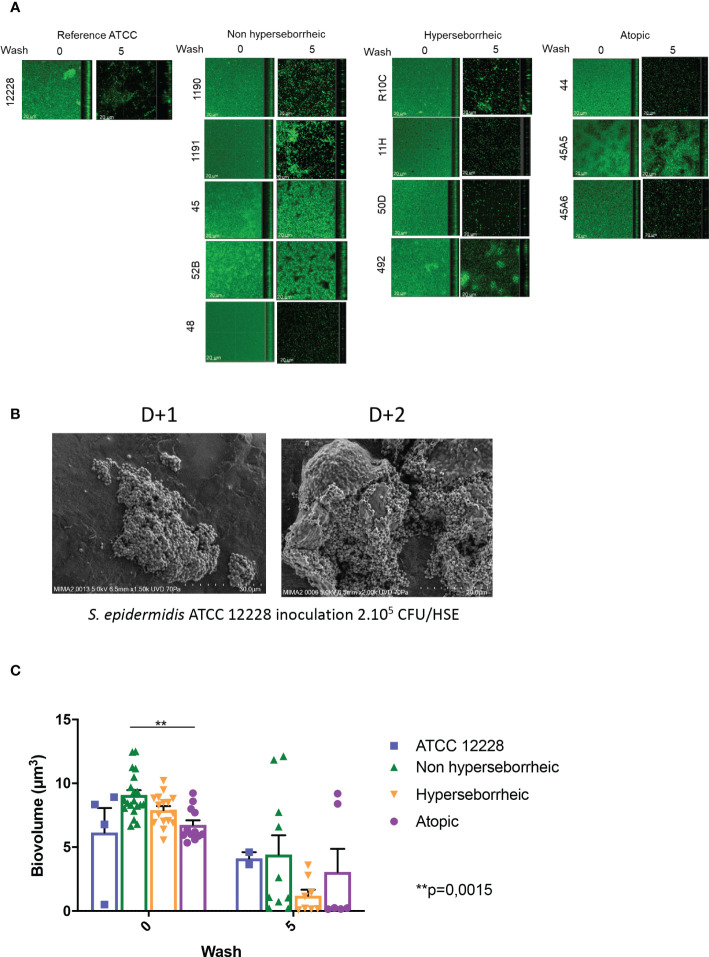
All *S. epidermidis* strains tested have the capacity to form biofilm. The biofilms formed on polystyrene were observed after 3 days of culture by confocal microscopy, before and after 5 washes **(A)** and their biovolumes were measured **(C)**. Moreover, the ATCC 12228 can form biofilm on 3D reconstructed skin 2 days after inoculation of 2.10^5^ CFU/HSE **(B)**. For statistical comparisons, (*) indicates comparison of all versus all samples grouped by number of washes, **p <0.01.

To date a few reports have described the variation of biofilm formation among commensal *S. epidermidis* isolates (71). We characterized the biofilm phenotype of the 11 *S. epidermidis* isolates and ATCC 12228, using confocal laser scanning microscopy (CLSM) and image analysis of biofilms in 96 well plates. Confocal microscopic images showed that all tested isolates of *S. epidermidis* were able to form a biofilm in the conditions of our experiment (**Figure 2A**). The capacity of *S. epidermidis* ATCC 12228 strain to form a biofilm has been also verified on the 3D reconstructed skin LabSkin using scanning electron microscopy (**Figure 2B**).

Using confocal microscopy, the 3D structure of each biofilm formed in the 96 wells was reconstructed before and after five washes with sterile water, thus allowing to describe biofilm cohesion and resistance to mechanical shear stress (**Figure 2A** for images and **Figure 2C** for the associated biovolumes). Before wash, the biovolumes were homogenous (average range: 6 to 8.6 µm^3^) for all the bacteria. The isolates from atopic skins origin were producing significantly less biofilms (*p* val = 0.0015) than the ones formed by bacteria from NH skins. The biovolume of the biofilm formed by the H strains and the ATCC 12228 were intermediate. After 5 washes, the cohesive behavior of each bacteria was different and was not related to the initial biovolume before wash. Three isolates (45, 52B from NH healthy skin and 45A5 from AD skin) formed particularly cohesive biofilms with high resistance to rinses compared to ATCC 12228, while other isolates were almost completely removed (biovolume < 1 µm^3^ after rinses). Altogether these data suggest that the skin origin is associated with a different capacity to form a thick biofilm and isolates from atopic skin seem to have thinner biofilms. However, skins origin cannot help to predict the resistance of the biofilm.

### 3.3 Proteases activities of *S. epidermidis* isolates correlates with skin origin

**Figure 3:**
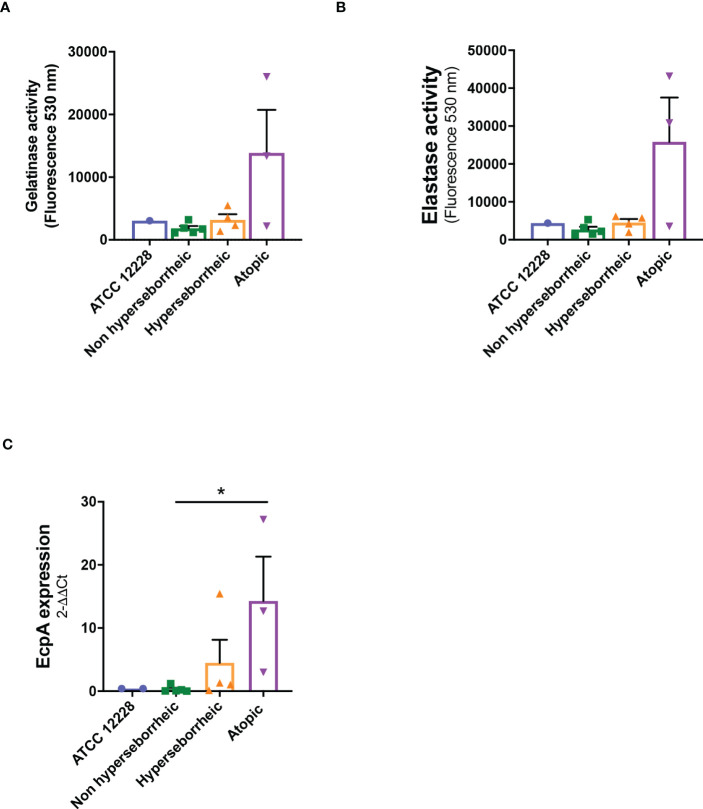
Live *S. epidermidis* from atopic skin can have a strong protease activity compared to strains from normal and hyperseborrheic skin. Average protease activity of the 3 groups of strains against gelatin-like **(A)** and elastase-like **(B)** measured in the supernatant of *S. epidermidis* cultured for 20h. **(C)** Average, for each skin type group, of the protease *EcpA* mRNA levels (normalized to *GyrB*) expressed after a co-culture of *S. epidermidis and* keratinocytes for 14h. For statistical comparisons, (*) indicates comparison of all versus all samples, *p <0.05.

The production of proteases by *S. epidermidis* has been recently highlighted as a potential virulence factor. More specifically EcpA, a cysteine protease, was shown to alter skin integrity, triggering inflammation and disrupting the skin physical barrier (8) (11). We first identified the presence of EcpA active domain sequence in the genomes of the isolates (**Supplementary Figure S2C**). Then we determined for each isolate the protease activity using collagenases and gelatinase *in vitro* assays. The average protease activity of the isolates pertaining to each of the three groups of bacteria (NH, H and AD) is presented (**Figures 3A, B**). As isolate 48, from a non lesional AD skin, showed low protease activities, it was included in the group of NH healthy strains. isolates from atopic lesional skins showed the highest protease activities. While isolates from healthy NH and H skins showed low gelatinase or elastase activity. Quantification of EcpA gene expression on all isolates co-cultured with keratinocytes were in accordance with the *in vitro* assays on protease activity. The level of EcpA expression in isolates from AD skins was significantly higher than the expression of EcpA in isolates from healthy NH and H skins (**Figure 3C**). Combination of *in vitro* assays and RT-qPCR showed that *S. epidermidis* isolates from atopic lesional skins had stronger protease activities compared to all other isolates tested and might have a higher potential to alter skin structure (11).

### 3.4 IL-8 secretion and tissue structure maintenance depend on the skin type origin of the bacteria

**Figure 4:**
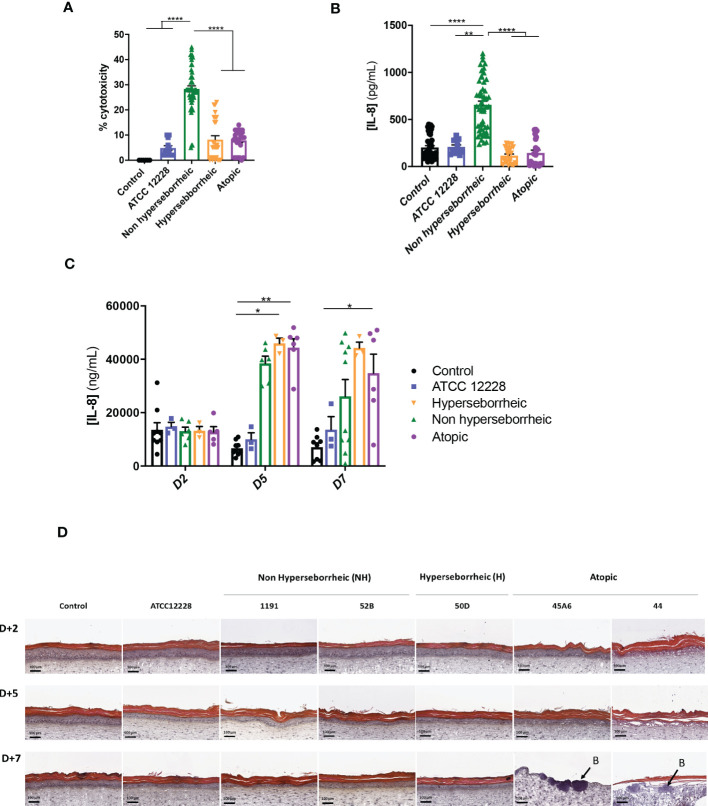
Live *S. epidermidis* effect on keratinocytes depends on skin type origin. **(A)** Average, for each skin type group of *S. epidermidis*, of the cytotoxicity and **(B)** IL-8 secretion in NHEK 2D co-culture and **(C)** IL-8 secretion in 3D reconstructed skin model after colonization. **(D)** HES images of tissues colonized with representative strains of the three skin types origins. Arrows indicate bacteria **(B)**. For statistical comparisons, (*) indicates comparison of all versus all samples, *p <0.05, **p<0.01, ****p<0.001.

In order to characterize further the interactions of theses isolates with the skin, *S. epidermidis* isolates were co-cultured for 14 hours with primary normal human epidermal keratinocytes (NHEK) and the effect of these isolates on cell viability and inflammatory cytokine IL-8 were analyzed. Observation of the cytotoxic effect of the bacteria, on the NHEK, showed a significant difference in association with the isolates’ origin. Bacteria isolated from NH healthy skins were significantly more cytotoxic than the ones isolated from H or AD skins (**Figure 4A**). For IL-8 production, co-culture with the bacteria isolated from NH skin leads to a significantly higher IL-8 release compared to the two others groups of isolates (**Figure 4B**), IL-6 and TNF-α were also quantified showing no significant modification between isolates but only a high level of TNF-α production in presence of the ATCC12228 strain (**Supplementary Figure S3**).

A test on reconstructed 3D skin model was then performed to characterize the effects on a skin tissue, using representative isolates of each group chosen based on their capacity to induce cytotoxicity and IL-8 production on NHEK: NH skin (1191, 52B), H skin (50D) and lesional AD skin (45A6, 44) as well as the reference strain ATCC 12228. Growth levels of the *S. epidermidis* bacteria on the model were quantified and presented similar ranges of CFU, except the reference strain that is slightly lower (**Supplementary Figure S4**). There was an increase in IL-8 secretion, from 5 days of colonization, with the clinical isolates; which was significantly higher than the reference strain ATCC 12228. Interestingly, the levels of IL-8 secretion measured were similar between the clinical isolates at each timepoint (**Figure 4C**). In contrast, histological observation of the colonized and non-colonized tissues showed remarkable differences between isolates (**Figure 4D** and **Supplementary Figure S5**). Specifically, colonization with isolates 45A6 and 44 from atopic lesional skin leads to a visible degradation of the viable epidermis and impacted the skin integrity, with the presence of bacteria in the dermal layer. Isolates from NH and H healthy skins had no visible impact on the epidermal morphology. It is important to note that these differences could not be linked to differences in growth levels on the model (**Supplementary Figure S4**). Altogether the data presented here showed distinct behavior of the *S. epidermidis* isolates depending on their skin origin and the type of the skin model. More particularly a higher cytotoxicity and IL-8 secretion were observed in the NHEK in co-culture with the bacteria isolated from NH skin, while they behave like the reference strain on the 3D differentiated model (**Supplementary Figure S5**). In contrast, bacteria isolated from AD lesional skin seemed to express pathogenic traits only on the more differentiated 3D model. As the reconstructed 3D model is more representative of the human skin as compared to NHK alone, we did not investigate further on the high cytotoxicity and IL-8 secretion in the 2D model.

### 3.5 Induction of AHR response pathway during interaction with *S. epidermidis* strains depends on the skin type origin of the bacteria

The AHR pathway has been described as implicated in several processes related to the skin immunomodulation and differentiation, through tryptophan derived ligands produced by UV light (FICZ) or microbial metabolism (indoles). Considering the potential of *S. epidermidis* to activate AHR, and the differential impact of the 12 isolates on skin cell cytotoxicity and inflammation, we investigated the effect of *S. epidermidis* isolates from different skin origins on this pathway. In order to follow *AHR* gene regulation in NHEK in co-culture with *S. epidermidis* strains, we quantified by qRT-PCR the expression of four genes: *CYP1A1, OVOL1, OVOL2* and *STAT6*. *CYP1A1* expression level is usually monitored to account for AHR induction in several cellular models. However, in keratinocytes other effectors are implicated like the couple OVOL1 and OVOL2; OVOL1 being linked to keratinocytes differentiation and OVOL2 keratinocytes proliferation, thus the balance between those two transcription factors may partially orient the cell maturation (72). STAT6, another transcription factor may be also involved in cell maturation through its inhibition of *OVOL1* expression (35).

**Figure 5:**
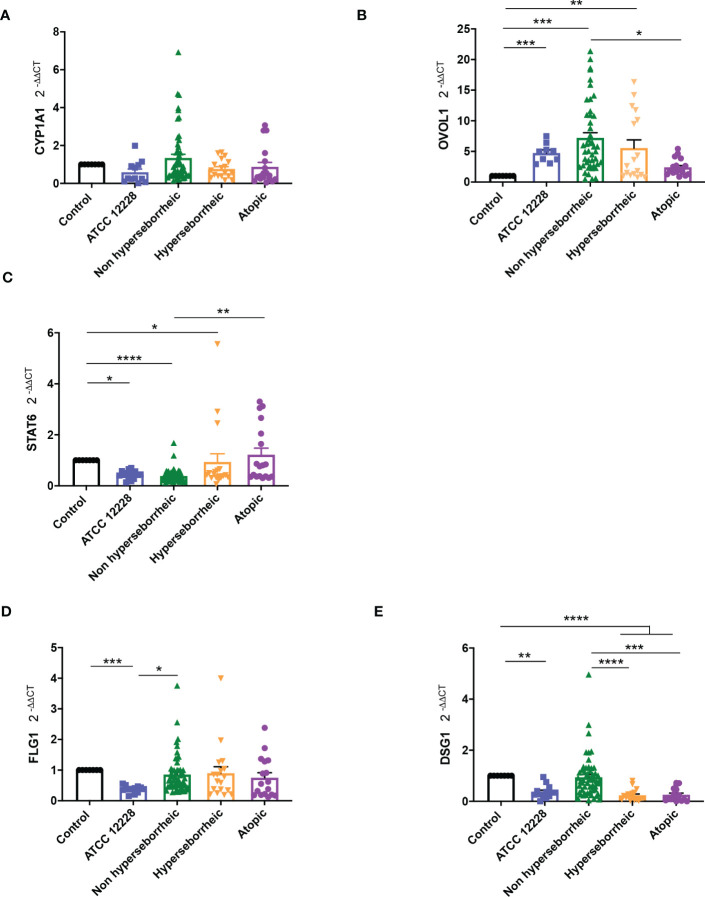
Live *S. epidermidis* can induce AhR pathway depending on skin type origin. Average of mRNA levels of AhR pathway genes CYP1A1 **(A)**, OVOL1 **(B)**, STAT6 **(C)** and average of mRNA levels of FLG1 **(D)** and DSG1 **(E)**, for each skin type group of *S. epidermidis*. For statistical comparisons, (*) indicates comparison of all versus all samples, *p <0.05, **p<0.01, ***p<0.005, ****p<0.001.

Results showed that *CYP1A1* was slightly induced, although not significantly, with the bacteria isolated from NH skin and that bacteria from AD or H skins had no effect on *CYP1A1* expression (**Figure 5A**). For NH skin associated strains, *OVOL1* expression followed the same pattern as *CYP1A1* as expected, knowing the regulation in the AHR but here the differences were statistically significant (**Figure 5B**). Isolates from H skin increased also significantly *OVOL1* expression. Interestingly, *STAT6* was inversely modulated compared to *CYP1A1* and *OVOL1* (**Figure 5C**) which follows the model of gene regulation described to date (35, 73). *OVOL2* expression was slightly decrease for all the bacteria (**Supplementary Figure S6A**). We also quantified filaggrin (*FLG*) expression, which is related to AHR/OVOL1 activation. Filaggrin is also a major protein involved in late differentiation of the epidermis (74). *FLG* expression levels obtained here did not allowed us to confirm a link with AHR pathway modulation for most of the *S. epidermidis* isolates tested. All the isolates maintained the same expression levels as the control (**Figure 5D**) except ATCC 12228 that showed a significant inhibition of *FLG* mRNA level. To address the skin integrity modifications observed in the 3D models, we also investigate Desmoglein 1 (*DSG1*) expression. Interestingly, *DSG1* expression followed the same pattern as *CYP1A1* (**Figure 5E**).

Following the expression of various effectors of the AHR regulation we showed a differential modulation of the AHR pathway depending on the skin origin of the *S. epidermidis* isolates. More particularly *S. epidermidis* bacteria isolated from NH healthy skin had an opposite effect on skin cell compared to those isolated from lesional AD skin, clear up-regulation of the AHR pathway in presence of isolates from NH healthy skin.

### 3.6 AHR ligands metabolites produced by *S. epidermidis* when interacting with NHEK

**Figure 6:**
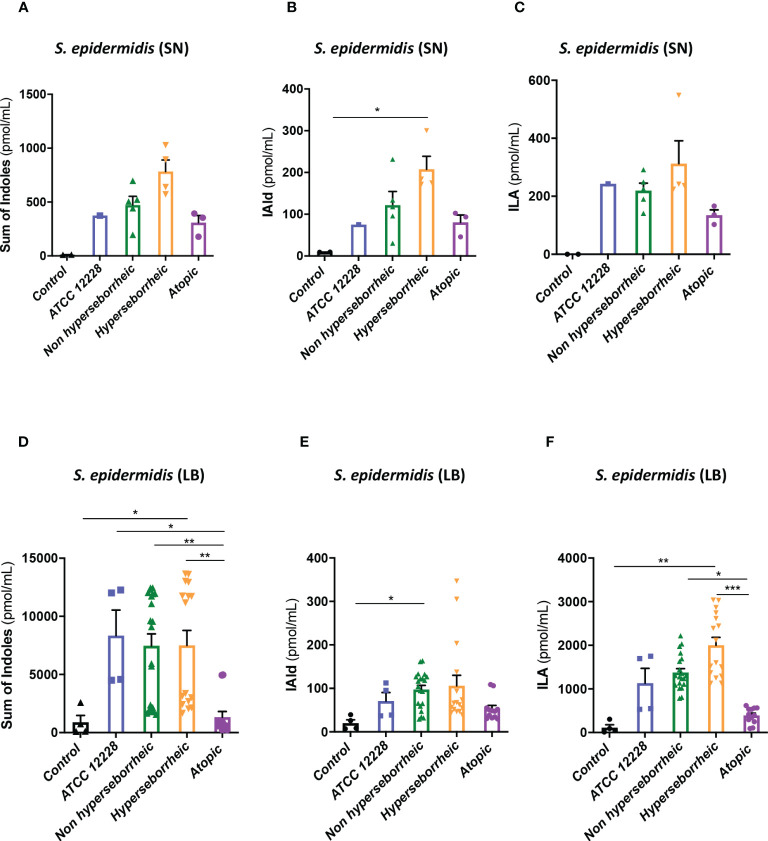
*S. epidermidis* produce indoles depending on skin type origin. **(A)** Sum of indoles (tryptamine + IAld + ILA + IAA) measured in the supernatant of *S. epidermidis* ATCC 12228, *S. epidermidis* from normal, hyperseborrheic or atopic skin type; quantification of Iald **(B)** and ILA **(C)** released in the culture medium. **(D)** Sum of indoles measured in the co-culture medium of NHEK and *S. epidermidis* ATCC 12228, *S. epidermidis* from normal, hyperseborrheic or atopic skin type; quantification of Iald **(E)** and ILA **(F)**. LB, Live bacteria; SN, supernatant. For statistical comparisons, (*) indicates comparison of all versus all samples, *p <0.05, **p<0.01, ***p<0.005.

**Figure 7:**
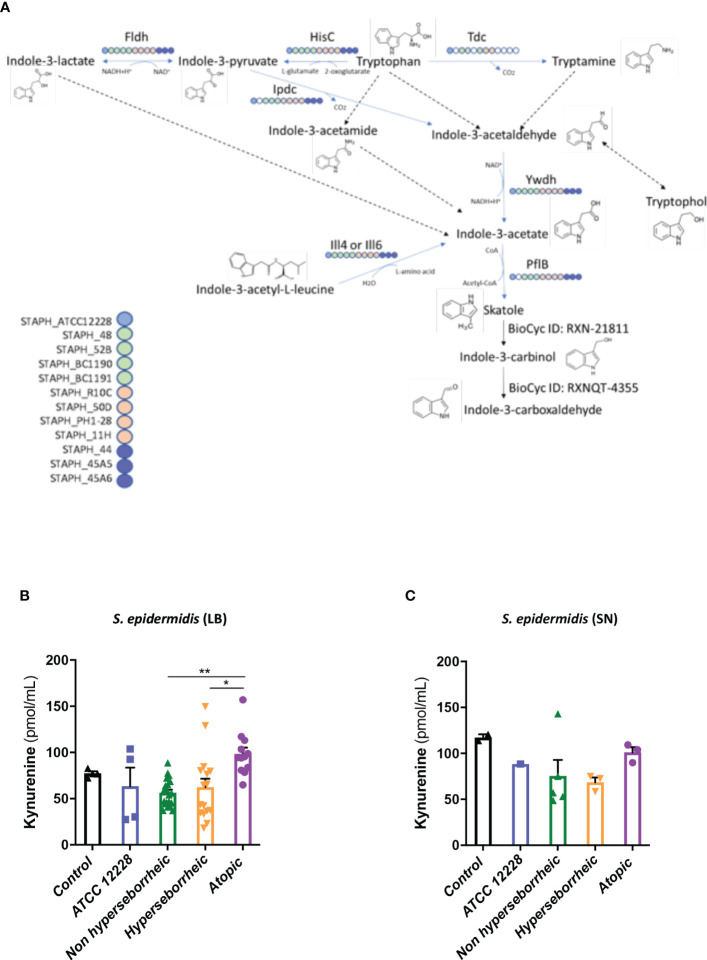
**(A)**: Reconstruction of tryptophan metabolic pathway in the 11 *S. epidermidis and* ATCC 12228, based on the genome sequences. Circles represent each strains: light blue ATCC 12228; green: non hyperseborrheic skin; orange: hyperseborrheic skin; dark blue: AD strains. Open circle means that the protein is not found. IAA, Indole Acetic Acid; IA, Indole-3-acetate; IAld, Indole-3-aldehyde; ILA, Indole-3-Lactic Acid. **(B, C)** Kynurenine metabolite quantification in the 11 S. epidermidis and the reference strain ATCC 12228 culture supernatant, after NHEK treatments with either living bacteria (LB) or bacterial supernatant (SN). Quantities are presented per group of skin type. For statistical comparisons, (*) indicates comparison of all versus all samples, *p <0.05, **p<0.01.

To better understand the interaction between *S. epidermidis* and AHR, we investigated the capacity of the 12 *S. epidermidis* isolates tryptophan derived metabolites. Moreover, a recent study demonstrated that AD patients have significantly lower level of indole-3-aldéhyde (IAld) than healthy subjects (31) on their skin. Tryptophan is the main precursor of AHR ligands produced by micro-organisms, allowing the biosynthesis of indoles ligands like indole-3-aldehyde (IAld), tryptamine, indole-3-lactic acid (ILA) or indole -3-acetic acid (IAA). In order to further understand the AHR regulation we analyzed the different AHR ligands produced by these bacteria cultivated alone or in co-culture with NHEK. We first quantified the total indoles produced by the bacteria cultivated in the same culture medium as for co-culture (KSFM) (**Supplementary Table T4**). This analysis showed that all *S. epidermidis* have the capacity to produce indoles and that isolates from atopic skins seemed to produce slightly lower quantities of indoles compared to the other two groups although the differences were not significant when looking at the sum of all indoles (tryptamine + IAld + ILA + IAA) (**Figure 6A**). Quantification of specific AHR ligands revealed that IAld and ILA, which is a precursor of IA, were following the same pattern as the total Indole with a lower production of these molecules in bacteria isolated from AD skins (**Figures 6B, C**). Higher levels of ligands (mainly IAld) were obtained with bacteria isolated from H skin. As *S. epidermidis* bacteria might not have the same metabolism when they are cultivated alone, or in co-culture with keratinocytes, we have quantified AHR ligands and precursors in the culture medium, after co-culture of *S. epidermidis* bacteria with NHEK (**Supplementary Table T5**). We observed again a lower production of indoles when we analyzed the supernatants of the co-culture of *S. epidermidis* bacteria isolated from AD skin with NHEK, as compared to the other two groups of isolates (**Figures 6D–F**). We explored the link between the differences in metabolites levels measured between the group of isolates, and the presence of predicted proteins in the indoles biosynthetic pathway of the *S. epidermidis* isolates (**Figure 7A**). Specifically, the enzyme Tdc (Aromatic-L-amino-acid decarboxylase; UNIPROT Q5HKV0) which converts tryptophan into tryptamine, a precursor of IA and IAld, is absent from the four genomes of AD isolates (lesional and non lesional), while this protein was predicted to be present in isolates from NH (52B, 1190) and H skin (R10C, 50D), supporting the lower levels of indoles quantified. Interestingly, kynurenine, a compound mainly produced by host cells through the IDO pathway, was significantly more produced when bacteria isolated from AD skin were incubated with NHEK, compared to other isolates (**Figure 7B**) while bacteria alone did not show differences on kynurenine production (**Figure 7C**).

In a tentative way of generalizing our results to more *S. epidermidis* isolates we used the public curated genomes sequences available in the literature in order to characterize them regarding the different genes implicated in the tryptophan metabolic pathways. 811 strains from the study by Zhou et al. were used as references: in their work Zhou and co-workers defined 10 clades using the genomes sequences. From these data, we constructed a heatmap of positive isolates in each clade for each gene implicated in indole metabolites biosynthesis pathway (**Supplementary Figure 7A**) and we rebuild a cladogram of these isolates to localize each of our isolates within these clades (**Supplementary Figure 7B**). From these data we were only able to confirm that concerning the *tdc* gene, which was absent in all AD isolates, the comparative genomic analysis supported in part these results, showing that most of the healthy isolates from our study are phylogenetically close to clade C which is enriched in *tdc* (59% of the isolates).

In order to confirm the link between AHR ligand production and AHR activation in NHEK, we used an inhibitor of AHR during the co-culture. The presence of AHR inhibitor had a deleterious effect on keratinocytes viability making impossible the analysis of keratinocytes gene expression. Finally, the data presented here showed that the 12 isolates have the capacity to produce AHR ligands in various ranges. Bacteria isolated from AD skin produce the lowest levels of indoles, which might explain the modulation of AHR pathway genes expression.

## 4 Discussion

**Figure 8:**
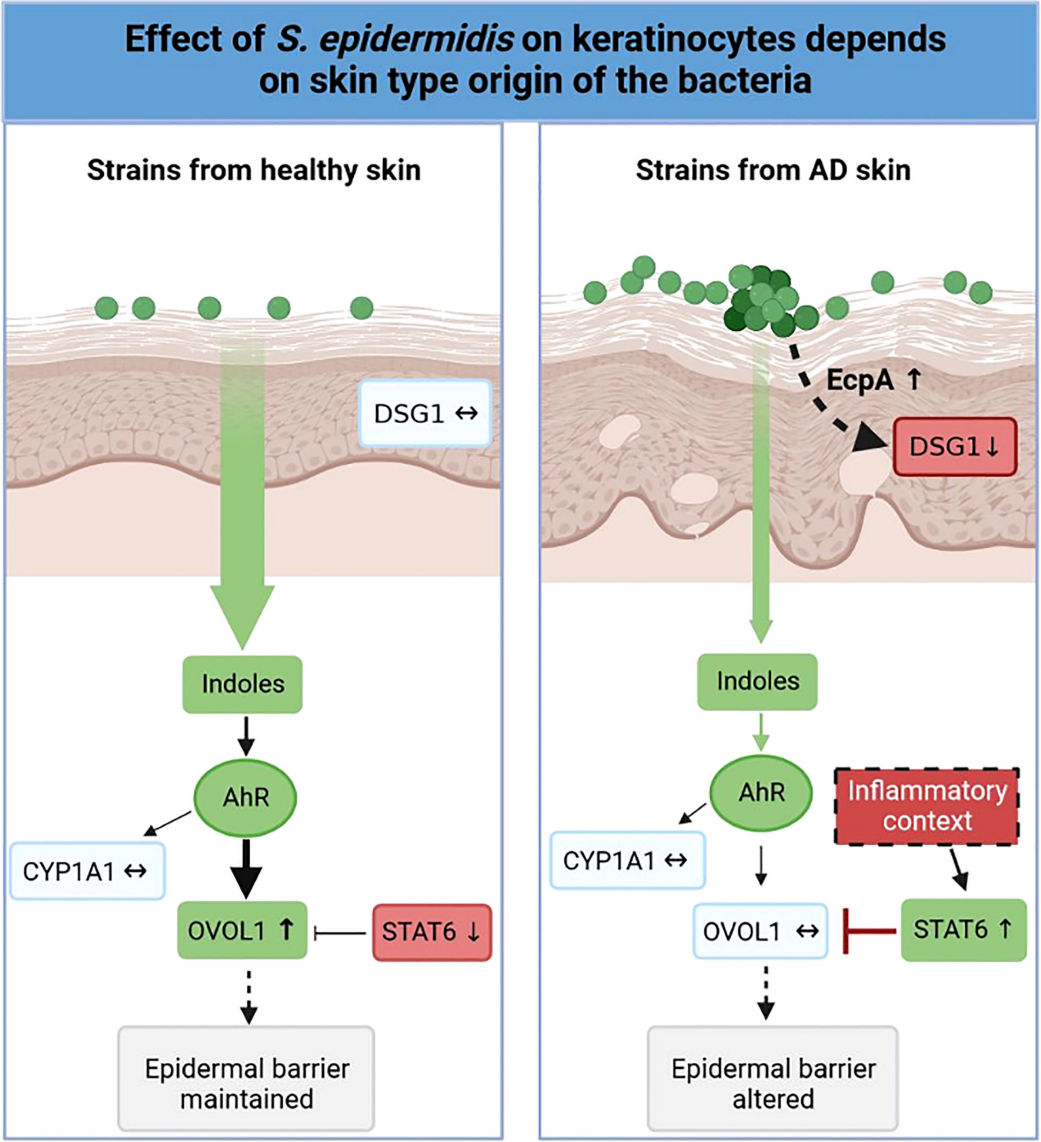
Graphical abstract. Effect of *S. epidermidis on* keratinocytes depends on skin type of origin of the bacteria. Strains from normal skin induce AhR/OVOL1 pathway via production of indoles while atopic strains produce low amounts of indoles and an overexpression of STAT6 resulting in a strong decrease of OVOL1 expression compared to normal and hyperseborrheic strains inductions. Moreover, strains from atopic skin induce tissue structure damages correlated with a strong protease activity and a decrease of DSG1. The thickness of the arrows represents the intensity of the induced signal, the dotted arrows represent links previously described in the literature but not verified in this study.

Variations in the diversity of the microbiota and predominance of specific strains in disease is well documented (19, 75–83), but the variability of the same species, in our case *S. epidermidis* isolated from different skin types (NH, H and AD) remains to be described. In this study, we demonstrated that isolates from NH and H healthy skins differed from AD skin. First at the genetic level, the genomes of the AD isolates clustered distinctly from bacteria isolated from NH and H skin. A more thorough analysis will be needed in order to identify whether these global differences could be pinpointed to specific genes or operons. At the phenotypic level, isolates from NH and AD skin presented contrasted proteases activities, AHR activation and indoles secretions, which were associated with the maintenance or not of the epidermis structure (**Figure 8**). Isolates from H skin had main characteristics in common with isolates from NH skin, however there were some minor differences at the level of indole secretion (ILA and IAld) and *DSG1* expression maintenance.

### 4.1 Biofilm thickness production is correlated to the origin skin type

Because previous studies comparing bacteria from different skin types (acne and healthy) have identified genetic variations in genes implicated in biofilm formation (25), we tested the ability of 12 different isolates of *S. epidermidis* to form a biofilm. Although *S. epidermidis* ATCC 12228 is independently described as a biofilm- or a non-biofilm-forming strain (67), we verified this capacity in our conditions and observed the formation of a thick biofilm both on a plastic support in a culture plate and on the LabSkin model emphasizing the importance of culture conditions for *S. epidermidis* biofilm formation (temperature, carbon sources, iron availability) (84, 85). All clinical isolates formed a biofilm *in vitro*, thus our results confirmed the capacity of *S. epidermidis* to form biofilms. This capacity is generally described in a pathogenic context (64, 86, 87), in contrast, here we demonstrated that isolates from NH skin form significantly bigger biofilms than isolates from AD skin. As a tentative way of explaining these differences between isolates, we tried to correlate with the presence/absence of genes known to be involved in biofilm formation (n = 28 as reported by Otto [6]) with the skin origin. The genes were retrieved in a very similar pattern among the isolates, and we did not observed differences based on the skin origin (**Supplementary Table T6**). In addition, when the gene was present in the *S. epidermidis* genome annotation (11/12 genomes), we compared the CDS organization of the accumulation-associated protein (*aap*), a protein involved in adhesion and biofilm formation [6] (88). However, all isolates harbored an *aap* gene coding for very similar proteins (**Supplementary Figure S2B**) and the differences in the CDS organization did not correlate with the skin origin. This suggested that the genes reported to be responsible for biofilm formation in *S. epidermidis* and specifically Aap may not be the reason of the phenotypic differences.

These data would need further investigation in 3D model in order to correlate immersed biofilm on plastic to biofilm growth on 3D reconstituted skin. It would help us understand whether this biofilm feature could be of biological importance for protection of the skin against other bacteria or environmental assaults as described in other contexts (detoxification in human gut (89); bioremediation (90)), and thus if this trait of isolates from NH skin might be of importance in skin physiology.

### 4.2 Proteases production is a key element to define *S. epidermis* effect on the skin

A recent study has shown that in the context of AD, the overgrowth of *S. epidermidis* could induce skin damage via protease EcpA that can disrupt the skin barrier and degrades desmoglein 1 (DSG-1) a key protein for physical barrier integrity (11). The same authors reported that this effect was dependent on the bacterial load. Here we add that EcpA expression is also dependent on the skin type origin of the isolate. Using a 3D reconstructed skin model, we characterized the isolates phenotype and compared it to the data obtained in 2D keratinocytes co-culture model. This allowed us to show that keratinocytes from the 3D model reacted very differently compared to the ones in 2D, with a more homogenous production of IL-8 among the isolates and no cytotoxicity induced by the bacteria from healthy skin (NH and H). Interestingly, the ATCC 12228 strain behaved in a very different way suggesting that this model strain is phenotypically far from the clinical isolates tested. This result needs to be considered when interpreting experiments done with bacterial strains in general in different *in vitro* models (59, 85–88, 91, 92). Additionally, the study of the histology pictures showed that the isolates from AD skin were not only producing more elastase-like, collagenase-like and EcpA proteases but their growth on the skin was much more deleterious than the 2 other type of strains with a clear degradation of the skin structure after one week of colonization. It can be hypothesized that this higher proteolytic activity is likely to explain in part why these isolates have such an effect on skin structures. Specific extraction of mRNA of these isolates under these conditions or the use of antiproteases could help us investigate this possibility. This suggests that bacteria isolated from AD skin, even if they belong to the same species as *S. epidermidis* from healthy skin, present specific phenotypic traits, which were captured using a differentiated human skin equivalent model.

### 4.3 AHR pathway and skin differentiation

*S. epidermidis* was described to active AHR pathway to mediate immune defenses (29) and activation of the AHR pathway is known to accelerate terminal differentiation by inducing an increase in the production of epidermal barrier proteins *in vitro* (keratinocytes from mice and humans) and *in vivo* (mice model) (93–94). Here, we have shown that AHR pathway could be induced by *S. epidermidis* as demonstrated by the *OVOL1* induction by strains from healthy skin. Additionally, *CYP1A1* and *DSG1* expression showed the same expression profile. A correlation between AHR activation, using chemical compound or bacterial indoles, and junctions proteins (Zonuline *ZO*, Claudine, Desmoglein 1 *DSG1*) over-expression, has been described in other cell types such as primary mammary epithelial cells (MECs) and enterocyte cells, however this link remains to be investigated in detail in skin cells (95, 96).

Once again, the effects were dependent on the skin type origin of the isolate, those from NH healthy skin induce *OVOL1* whereas strains from atopic skin induce *STAT6*. In the context of AD, it was recently shown that the high level of IL-4 and IL-13 induce the decrease of FLG-1 via induction of STAT6 (73). Thus, we can speculate that isolates from AD skin could inhibit the epidermal differentiation induced by AHR by activating the inhibitor STAT6 whereas isolates from NH healthy skin participate in epidermal differentiation by activating the OVOL1 axis, but there is still need of experimental data to fully confirm such work hypothesis.

When following the product of AHR pathway it was remarkable to show that levels of ligands produced by the isolates from H skin did not correlate with any expression markers or production compared to the other isolates suggesting a lack of knowledge on the AHR regulation is still important. Additionally, it was clear that the AHR ligands secreted when the bacteria were alone in the medium or in interaction with keratinocytes were different in quantities but also the pattern between isolates were different. Interestingly a strong correlation was observed when we compared the pattern of ligands production in co-culture with the OVOL1 axis. This suggest that another level of complexity is added to the comprehension of AHR pathway regulation since the bacterial environment seems to affect strongly the ligands secretion. This result suggests the need of an interaction between the bacteria and the keratinocytes, but the mechanism is not clear. The bacteria could secrete AHR ligands, with different capacity to induce AHR pathway, depending on its environment and interactions with cells. For example, the indole production in *E.coli* depends on pH, temperature and the presence of antibiotics in the medium (97). Considering a study showing that co-culture of bacteria (*Pseudomonas* sp. and *Streptomyces* sp.) led to the upregulation of indoles that were not previously observed in the monocultures of each bacteria (98), the ability of *S. epidermidis* from healthy skin to produce indoles might be potentiated by the addition of other bacteria or fungi from healthy skin microbiota. For instance, it could be interesting to mix *S. epidermidis* with *Malassezia* which can induce AHR pathway by secreting malassezin (99) and observe the expression of differentiation markers by keratinocytes. Additionally, the role of the keratinocytes themselves is open to question, would they consume some AHR ligands more than other? The complexity of the events that can occur when *S. epidermidis* and keratinocytes are co-cultured plead for further experiments in order to disconnect each event of this interaction and how such modulation of AHR pathway may impact on skin maturation.

Genomics analysis using public genome databases on 811 genomes of *S. epidermidis* strains and targeting the genes involved in the AHR pathway showed that the very heterogenous pattern of *tdc* gene presence/absence between the different clades suggested that this gene might be an interesting target to define *S. epidermidis* isolates diversity.

## 5 Conclusions

In conclusion, this study first confirmed that the role of *S. epidermidis* in epidermal differentiation could be partly mediated by its ability to regulate AHR signaling but also led us to postulate that the capacity of the isolates to secrete indoles which in turn activate the AHR pathway might be dependent of the skin type of origin of the bacteria. These results are consistent with current understanding of the interactions between microbiota and healthy skin or the pathogenesis of AD (7, 35, 100). However, the AHR pathway is differently regulated by many ligands with synergic or opposite effects and further work, involving a larger number of isolates and full bacterial communities, is needed to understand the modulation of this pathway in health and disease by the microbiota.

## Data availability statement

The datasets presented in this study can be found in online repositories. The names of the repository/repositories and accession number(s) can be found below: https://www.ncbi.nlm.nih.gov/, PRJNA721837 https://www.ncbi.nlm.nih.gov/, PRJNA721841 https://www.ncbi.nlm.nih.gov/, PRJNA721842 https://www.ncbi.nlm.nih.gov/, PRJNA721843 https://www.ncbi.nlm.nih.gov/, PRJNA721840 https://www.ncbi.nlm.nih.gov/, PRJNA662445 https://www.ncbi.nlm.nih.gov/, PRJNA721839 https://www.ncbi.nlm.nih.gov/, PRJNA721845 https://www.ncbi.nlm.nih.gov/, PRJNA721847 https://www.ncbi.nlm.nih.gov/, PRJNA721846 https://www.ncbi.nlm.nih.gov/, PRJNA721836.

## Ethics statement

The studies were conducted in compliance with the 1975 World Medical Association Declaration of Helsinki, national and EU regulations and L’Oréal Research and Innovation’s procedures based on ICH guidelines for Good Clinical Practice. For study ACR/COPEG/1125 (2013, France), according to the national “Arrêté du 11 mai 2009 relatif aux définitions de certaines catégories de recherches biomédicales”, this non-interventional study without tested product nor invasive assessment method did not require Regulatory Approval. However, this study has been approved by L’Oréal’s Ethic’s Group. For the cohort 2 (2012, France), the study was approved by the DOST ethical committee. All volunteers received verbal and written information concerning the study in accordance with the applicable local regulations, guidelines and the current SOP. This information explained the nature, purpose and risks of the study and emphasized that participation in the study was voluntary and that the volunteer might withdraw from the study at any time and for any reason. The volunteer’s written informed consent to participate in the study was obtained prior to any study related procedure being carried out. All data was analyzed anonymously and steps were taken to protect the identities of all participants, according to national law “Informatique et liberté” dated January 6th 1978, modified by law No 94–548 dated July 1st 1994, and law n° 2004–801 dated August 6th 2004, applicable at the time of the study.

## Author contributions

LL performed lab work, analyzed data, statistical analyses, and wrote the manuscript. GC, EF, CF performed lab work. JV and SM performed sequencing, bioinformatics and statistical analyses. MR conceive and supervised the study, performed bioinformatics analyses, statistical analyses, contribute to and wrote the manuscript. CC conceived and supervised the study, contribute to analyze the data and wrote the paper. RB, HS, M-LM, AG, DB, J-MC, LA and PL provided input on the analyses and writing of the manuscript. All authors contributed to the revision of the manuscript and have approved the final version of the manuscript.

## Supplementary material

Supplementary Figure 1 Phylogenetic tree of the 11 complete draft genomes of *S. epidermidis* using FastANI v1.31 (42) by calculating the Average Nucleotide Index for each genome pair. The tree was generated using FastTree v2.1.11 (43).

Supplementary Figure 2
**(A)** Comparison of the genomic structure of Stb20 and STB20-like phage loci identified in seven *S. epidermidis* strains. **(B)**: Accumulation-associated protein (aap) CDS organization in the 10 *S. epidermidis* strains. **(C)**: EcpA active domain comparison. Alignment of active amino acid sequences for the 12 *S. epidermidis* strains. *S. epidermidis* cysteine protease EcpA (UniProtKB/Swiss-Prot: P0C0Q0.1) were aligned using MAFFT multiple alignment tool.

Supplementary Figure 3 Live *S. epidermidis* effect on keratinocytes depends on skin type origin. **(A)** Average, for each skin type group of *S. epidermidis*, of IL-6 and TNF-alpha secretion in NHEK 2D co-culture. For statistical comparisons, (*) indicates comparison of all versus all samples, **p <*0.05, **p<0.01, ***p<0.005.

Supplementary Figure 4
*S. epidermidis* growth on LabSkin HSE model from D0 (inoculation) to D7 post inoculum. The reference strain ATCC 12228 is in blue, the strains from non hyperseborrheic skin in green (1191, 52B), the strain from hyperseborrheic skin in orange (50D) and the strains from atopic skin in purple (44, 45A6).

Supplementary Figure 5 Live *S. epidermidis* effect on keratinocytes depends on skin type origin. Magnification part of HES images of tissues colonized with representative strains of the three skin types origins.

Supplementary Figure 6 Average, per group of skin type, of OVOL2 mRNA expression *in* NHEK co-cultivated with one of the 11 *S. epidermidis* isolates or the reference strain ATCC 12228.

Supplementary Figure 7
**(A)**: Heatmap of positive isolates in each clade for each gene implicated in indole metabolites biosynthesis pathway; **(B)** cladogram to localize each of the 11 isolates within the previous described clades.
