# Supplementary material for: Staphylococcus epidermidis isolates from atopic or healthy skin have opposite effect on skin cells: potential implication of the AHR pathway modulation
Source: Front Immunol. 2023 May 26;14:1098160. doi: 10.3389/fimmu.2023.1098160 (PMC10250813; doi:10.3389/fimmu.2023.1098160)

Chromatogrammes

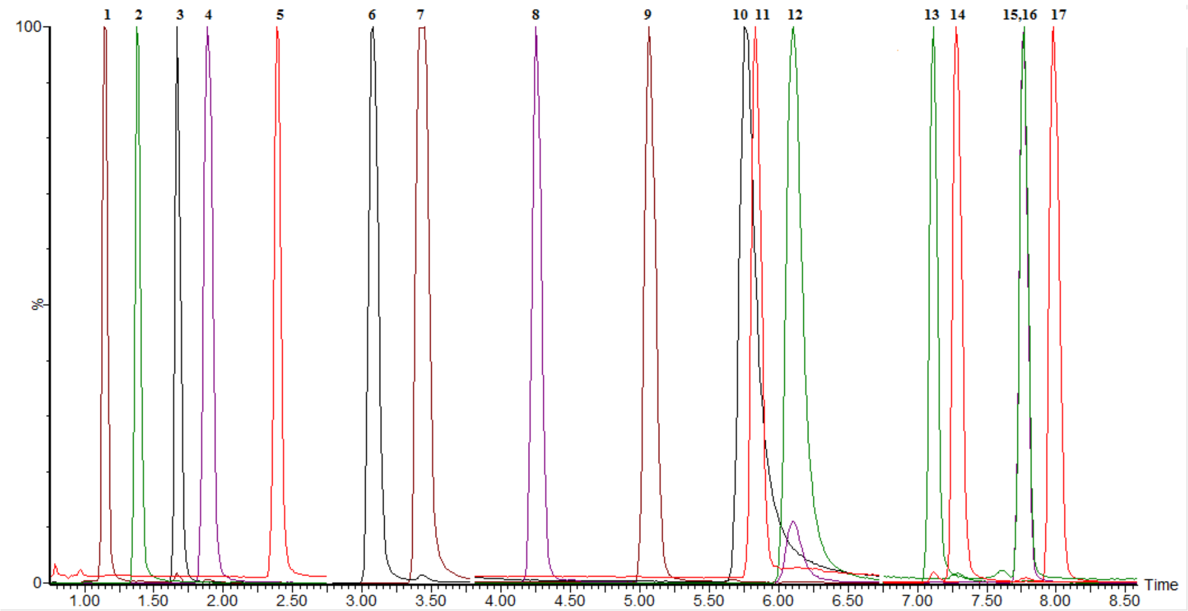

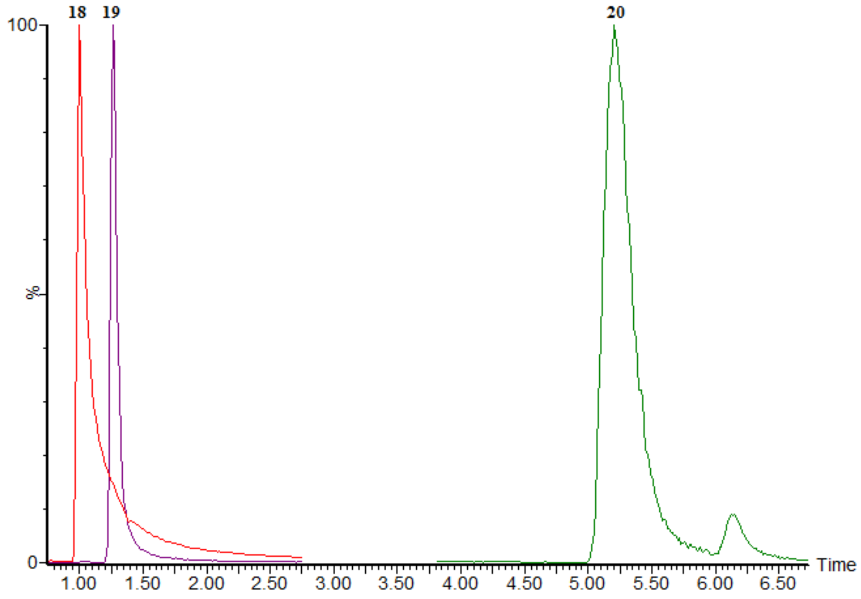

ID chromatogramme	Métabolites
1	3-OH-kynurenine
2	Serotonin
3	5-Hydroxytryptophane
4	Kynurenine
5	3-OH-anthranilic acid
6	Tryptamine
7	Tryptophan
8	5-Hydroxyindole acetic acid
9	N-acetyserotonine
10	Xanthurenic acid
11	Indole-3-acetamide
12	Kynurenic acid
13	Indole-3-lactic acid
14	Indole-3-aldehyde
15	Tryptophol
16	Indole-3-acetic acid
17	Melatonin
18	Picolinic acid
19	Quinolinic acid
20	Indoxyl-3-Sulfate

Calibration curves

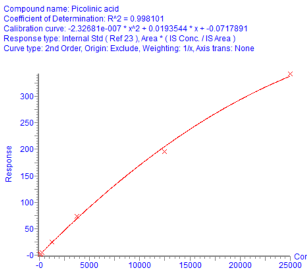

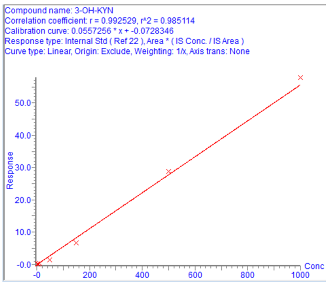

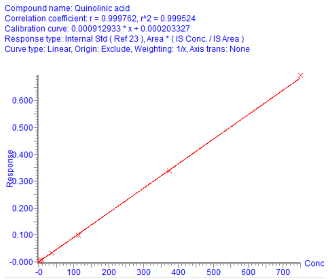

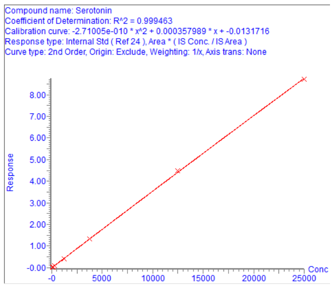

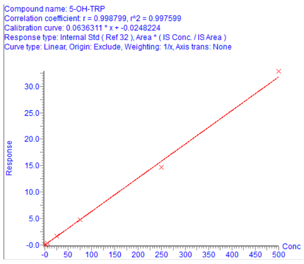

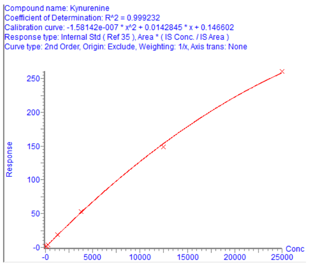

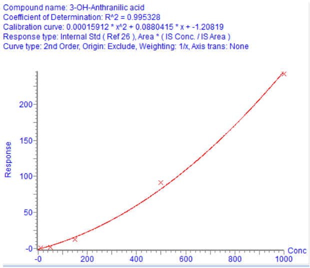

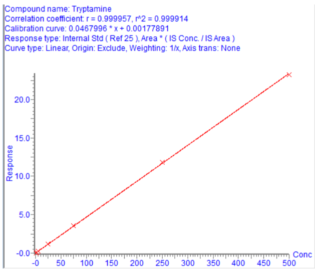

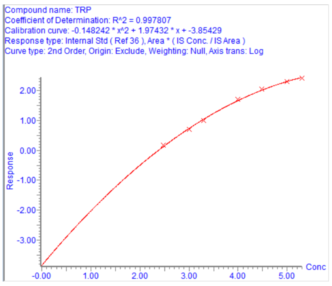

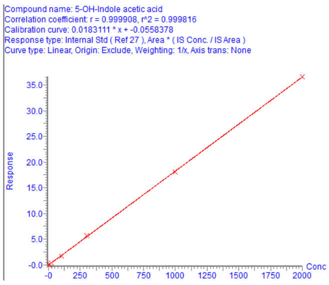

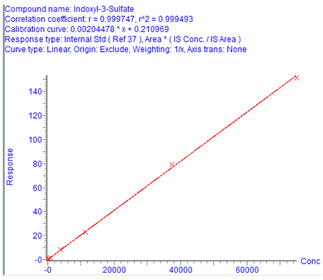

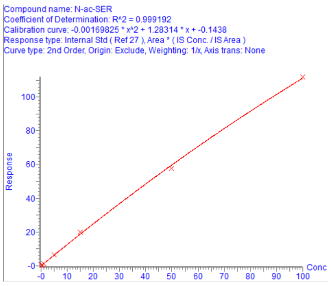

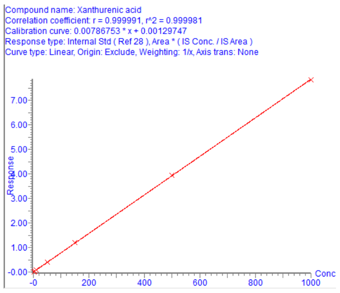

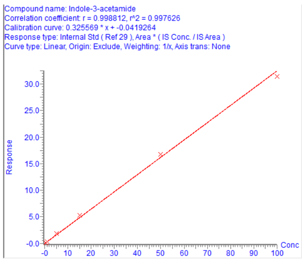

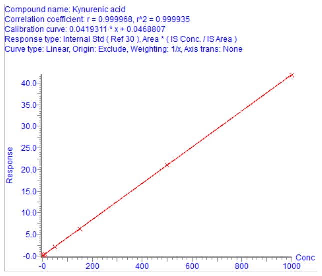

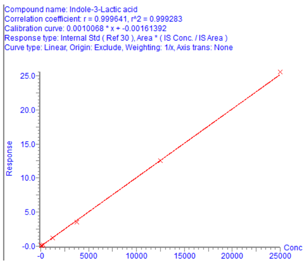

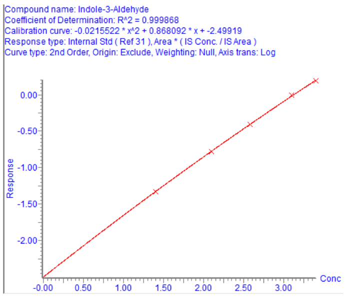

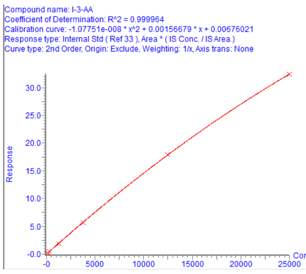

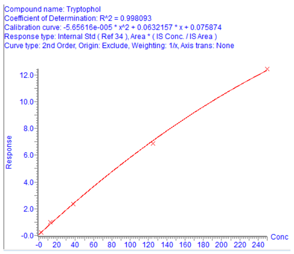

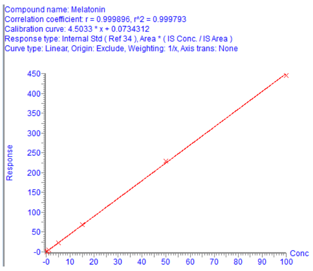

Supplement: Supplementary file 1 [file DataSheet_1.pdf]
