# Supplementary material for: Staphylococcus epidermidis isolates from atopic or healthy skin have opposite effect on skin cells: potential implication of the AHR pathway modulation
Source: Front Immunol. 2023 May 26;14:1098160. doi: 10.3389/fimmu.2023.1098160 (PMC10250813; doi:10.3389/fimmu.2023.1098160)
Supplement: Supplementary file 2 [file Table_1.pdf]

Strain		NGS	# reads	# bp	SRA seq files	Replicons	GenBank acc.	Size (bp)	Cov. (x)	RMBC	BioProject	BioSample
		Illumina_F	10 427 315	867 320 316		1 chromosome	CP090575	*2 405 813*		95.8%		
**11H**	PH1-4	Illumina_R	10 427 315	860 606 794	SRP354709	+ 3 plasmids		2 431 849	1389		PRJNA721837	SAMN18740291
		Nanopore	400 871	1 650 894 937						93.7%		
		Illumina_F	6 658 024	559 077 356		1 chromosome	CP090912	*2 389 522*		96.8%		
**44**	PH2-28	Illumina_R	6 658 024	554 386 207	SRP355409	+ 2 plasmids		2 401 799	2071		PRJNA721841	SAMN18740294
		Nanopore	908 900	3 861 742 514						99.4%		
		Illumina_F	5 635 592	468 316 215		1 chromosome	CP090915	*2 480 092*		97.1%		
**45A5**	PH2-10	Illumina_R	5 635 592	464 888 452	SRP355912	+ 6 plasmids		2 590 689	1192		PRJNA721842	SAMN18740295
		Nanopore	682 066	2 156 033 600						96.5%		
		Illumina_F	4 597 079	380 732 512		1 chromosome	CP090922	*2 439 442*		98.6%		
**45A6**	PH2-43	Illumina_R	4 597 079	377 958 393	SRP356087	+ 1 plasmid		2 447 700	3586		PRJNA721843	SAMN18740296
		Nanopore	1 974 349	8 019 320 611						97.8%		
		Illumina_F	6 335 272	527 048 682		1 chromosome	CP090924	*2 227 438*		63.6%		
**48**	PH2-35	Illumina_R	6 335 272	523 042 281	SRP356115	+ 4 plasmids		2 300 582	2120		PRJNA721840	SAMN18740293
		Nanopore	1 241 949	3 826 489 231						81.8%		
		Illumina_F	5 547 332	470 599 152		1 chromosome	CP066376	*2 468 349*		97.9%		
**492**	PH1-28	Illumina_R	5 547 332	465 502 785	SRP298503	+ 5 plasmids		2598183	5341		PRJNA662445	SAMN16085533
		Nanopore	1 265 187	12 939 878 179						99.9%		
		Illumina_F	6 299 842	534 195 240		1 chromosome	CP090985	*2 435 353*		99.1%		
**50D**	PH1-27	Illumina_R	6 299 842	528 916 613	SRP357527	+ 3 plasmids		2 494 613	2236		PRJNA721839	SAMN18740292
		Nanopore	1 119 374	4 515 537 291						98.8%		
		Illumina_F	7 396 196	616 324 203		1 chromosome	CP090989	*2 502 349*		94.6%		
**52B**	PH1-2	Illumina_R	7 396 196	610 929 616	SRP357528	+ 3 plasmids		2 532 862	3309		PRJNA721845	SAMN18740297
		Nanopore	1 917 950	7 153 133 491						94.5%		
		Illumina_F	7 262 971	603 391 624		1 chromosome	CP090993	*2 429 877*		97.3%		
**BC1190**		Illumina_R	7 262 971	598 593 569	SRP357541	+ 4 plasmids		2 521 740	1604		PRJNA721847	SAMN18740299
		Nanopore	746 849	2 843 865 779						98.8%		
		Illumina_F	5 680 641	471 432 108		1 chromosome	CP090998	*2 384 550*		97.2%		
**BC1191**		Illumina_R	5 680 641	468 094 273	SRP357603	+ 7 plasmids		2 464 714	1809		PRJNA721846	SAMN18740298
		Nanopore	871 895	3 519 243 538						97.8%		
		Illumina_F	8 442 557	701 543 160		1 chromosome	CP091006	*2 465 749*		97.4%		
**R10C**	PH1-18	Illumina_R	8 442 557	695 339 837	SRP357604	+ 4 plasmids		2 557 614	2566		PRJNA721836	SAMN18740290
		Nanopore	1 217 469	5 165 698 778						99.2%		

Supp. Table T1: Reads parameters obtained after Illumina sequencing and reads cleaning steps

C1 - Internal use
