# Supplementary material for: Staphylococcus epidermidis isolates from atopic or healthy skin have opposite effect on skin cells: potential implication of the AHR pathway modulation
Source: Front Immunol. 2023 May 26;14:1098160. doi: 10.3389/fimmu.2023.1098160 (PMC10250813; doi:10.3389/fimmu.2023.1098160)
Supplement: Supplementary file 3 [file Table_2.pdf]

STRAIN	REGION	REGION_LENGTH	COMPLETENESS(score)	MOST_COMMON_PHAGE_NAME(hit_genes_count)
11H	1	34.6Kb	incomplete(30)	PHAGE_Staphy_PT1028_NC_007045(7)
11H	2	18.5Kb	incomplete(20)	PHAGE_Staphy_PT1028_NC_007045(8)
11H	3	14.1Kb	incomplete(20)	PHAGE_Staphy_SPbeta_like_NC_029119(3)
11H	4	6Kb	incomplete(20)	PHAGE_Cyanop_Syn30_NC_021072(1)
11H	5	6.1Kb	incomplete(20)	PHAGE_Strept_Dp_1_NC_015274(2)
44	1	14.1Kb	incomplete(20)	PHAGE_Staphy_SPbeta_like_NC_029119(3)
44	2	6.1Kb	incomplete(20)	PHAGE_Strept_Dp_1_NC_015274(2)
45A5	1	57.7Kb	intact(150)	PHAGE_Staphy_StB20_NC_019915(28)
45A5	2	46.8Kb	intact(150)	PHAGE_Staphy_StB12_NC_020490(22)
45A6	1	5.8Kb	incomplete(30)	PHAGE_Bacill_SP_15_NC_031245(1)
45A6	2	43.5Kb	intact(150)	PHAGE_Staphy_StB20_like_NC_028821(31)
45A6	3	4.2Kb	incomplete(30)	PHAGE_Bacter_Diva_NC_028788(2)
48	1	35.3Kb	incomplete(30)	PHAGE_Staphy_PT1028_NC_007045(8)
48	2	41.5Kb	intact(120)	PHAGE_Staphy_StB20_like_NC_028821(42)
492	1	50.7Kb	intact(150)	PHAGE_Staphy_StB20_like_NC_028821(29)
492	2	8.7Kb	incomplete(40)	PHAGE_Bacill_G_NC_023719(1)
50D	1	98.3Kb	intact(150)	PHAGE_Staphy_StB20_like_NC_028821(32)
52B	1	49.7Kb	incomplete(50)	PHAGE_Bacill_vB_BhaS_171_NC_030904(7)
52B	2	30.9Kb	incomplete(50)	PHAGE_Staphy_PT1028_NC_007045(9)
TCC1222	1	63.7Kb	intact(150)	PHAGE_Staphy_SPbeta_like_NC_029119(4)
BC1190	1	43.5Kb	intact(150)	PHAGE_Staphy_StB20_like_NC_028821(31)
BC1191	1	6Kb	incomplete(20)	PHAGE_Cyanop_Syn30_NC_021072(1)
BC1191	2	6.6Kb	incomplete(20)	PHAGE_Arthro_Mufasa8_NC_049478(2)
BC1191	3	21Kb	incomplete(20)	PHAGE_Staphy_StauST398_4_NC_023499(1)
BC1191	4	10.1Kb	incomplete(20)	PHAGE_Staphy_SPbeta_like_NC_029119(3)
R10C	1	43.5Kb	intact(150)	PHAGE_Staphy_StB20_like_NC_028821(31)

Supp. Table T2: Phage sequences identified from the 12 genomes using PHASTER.
Intact sequences are highlighted in yellow.

C1 - Internal use
