# Supplementary material for: Staphylococcus epidermidis isolates from atopic or healthy skin have opposite effect on skin cells: potential implication of the AHR pathway modulation
Source: Front Immunol. 2023 May 26;14:1098160. doi: 10.3389/fimmu.2023.1098160 (PMC10250813; doi:10.3389/fimmu.2023.1098160)
Supplement: Supplementary file 4 [file Table_3.pdf]

Strain	*aadD*	*apH3'*	*blaI*	*blaR*	*blaZ*	*bleO*	*dfrC*	*fosB*	*fusB*	*fusC*	*mgrA*	*msrA*	*norA*	*rlmH*	*tetK*
BC1190	X	X	X	X	X	X	X	X			X	X	X		
R10C	X	X	X	X	X	X	X	X			X	X	X		
ATCC12228		X	X	X	X		X	X			X		X	X	X
BC1191		X	X	X	X		X	X			X		X	X	
45A6		X	X	X	X		X	X			X		X	X	
11H		X	X	X	X		X	X	X		X	X	X	X	
44		X	X	X	X		X	X			X		X		
50D		X	X	X	X		X	X			X		X		
45A5		X					X	X			X		X		
492		X					X	X			X		X		
52B		X					X	X		X	X		X	X	
48		X					X				X		X		

Supp. Table T3: Antimicrobial resistance genes identified in the genomes of the 12 *S. epidermidis* strains using ABRicate tool.

C1 - Internal use
