# Supplementary figures and images for: Staphylococcus epidermidis isolates from atopic or healthy skin have opposite effect on skin cells: potential implication of the AHR pathway modulation
Source: Front Immunol. 2023 May 26;14:1098160. doi: 10.3389/fimmu.2023.1098160 (PMC10250813; doi:10.3389/fimmu.2023.1098160)

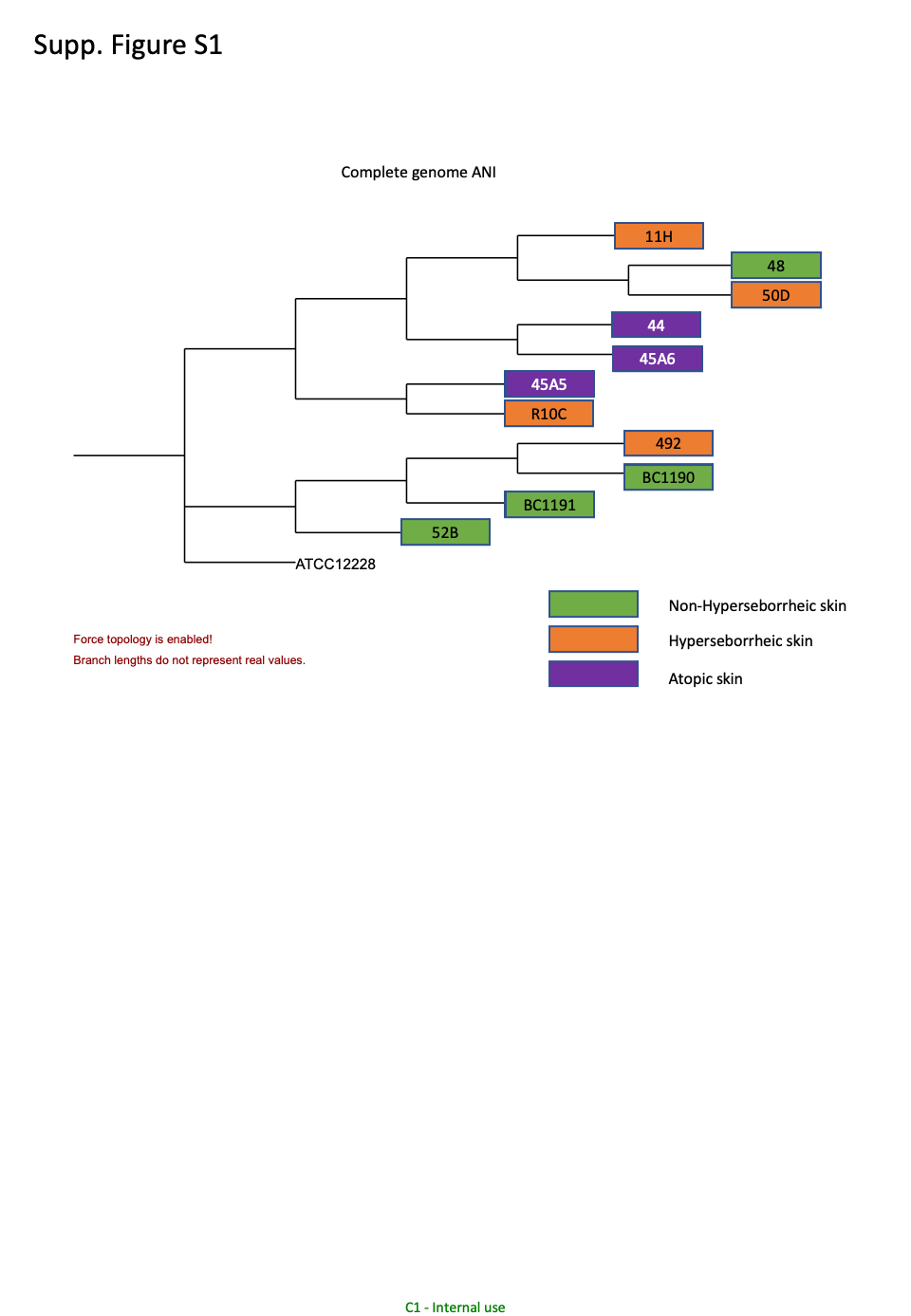

Supplement: Supplementary Figure 1 — Phylogenetic tree of the 11 complete draft genomes of S. epidermidis using FastANI v1.31 (42) by calculating the Average Nucleotide Index for each genome pair. The tree was generated using FastTree v2.1.11 (43). [file Image_1.tiff]

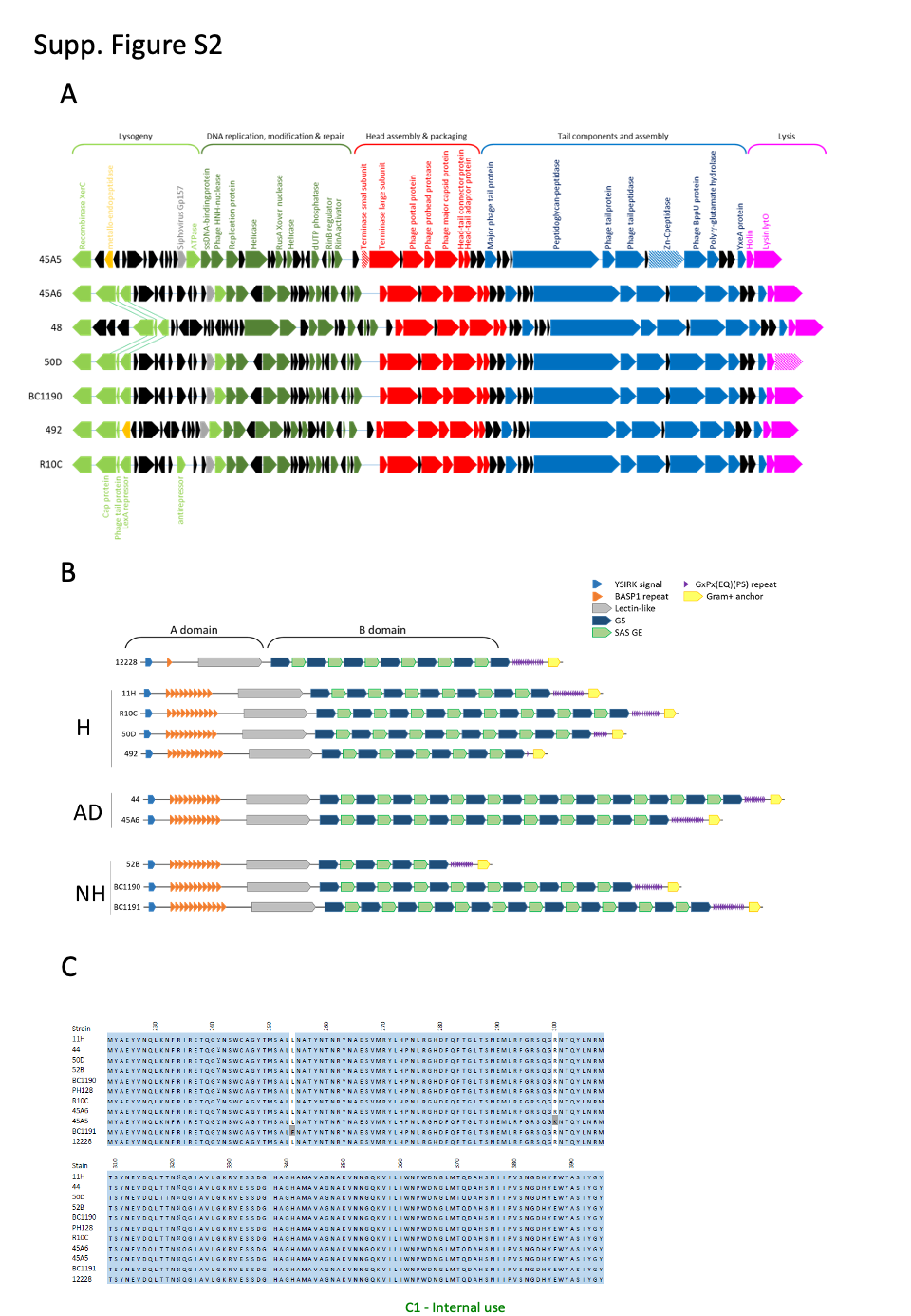

Supplement: Supplementary Figure 2 — (A) Comparison of the genomic structure of Stb20 and STB20-like phage loci identified in seven S. epidermidis strains. (B): Accumulation-associated protein (aap) CDS organization in the 10 S. epidermidis strains. (C): EcpA active domain comparison. Alignment of active amino acid sequences for the 12 S. epidermidis strains. S. epidermidis cysteine protease EcpA (UniProtKB/Swiss-Prot: P0C0Q0.1) were aligned using MAFFT multiple alignment tool. [file Image_2.tiff]

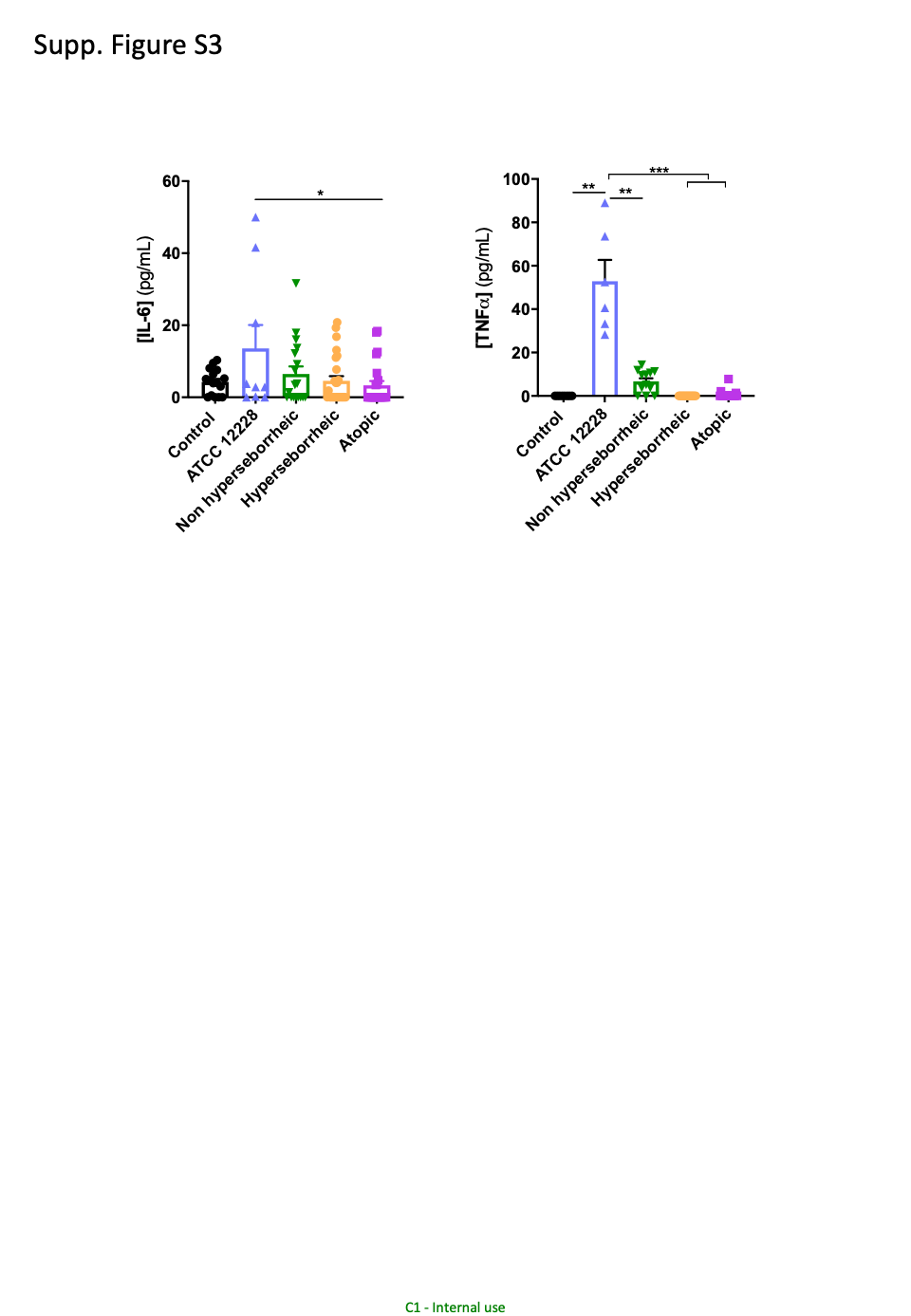

Supplement: Supplementary Figure 3 — Live S. epidermidis effect on keratinocytes depends on skin type origin. (A) Average, for each skin type group of S. epidermidis, of IL-6 and TNF-alpha secretion in NHEK 2D co-culture. For statistical comparisons, (*) indicates comparison of all versus all samples, *p <0.05, **p<0.01, ***p<0.005. [file Image_3.tiff]

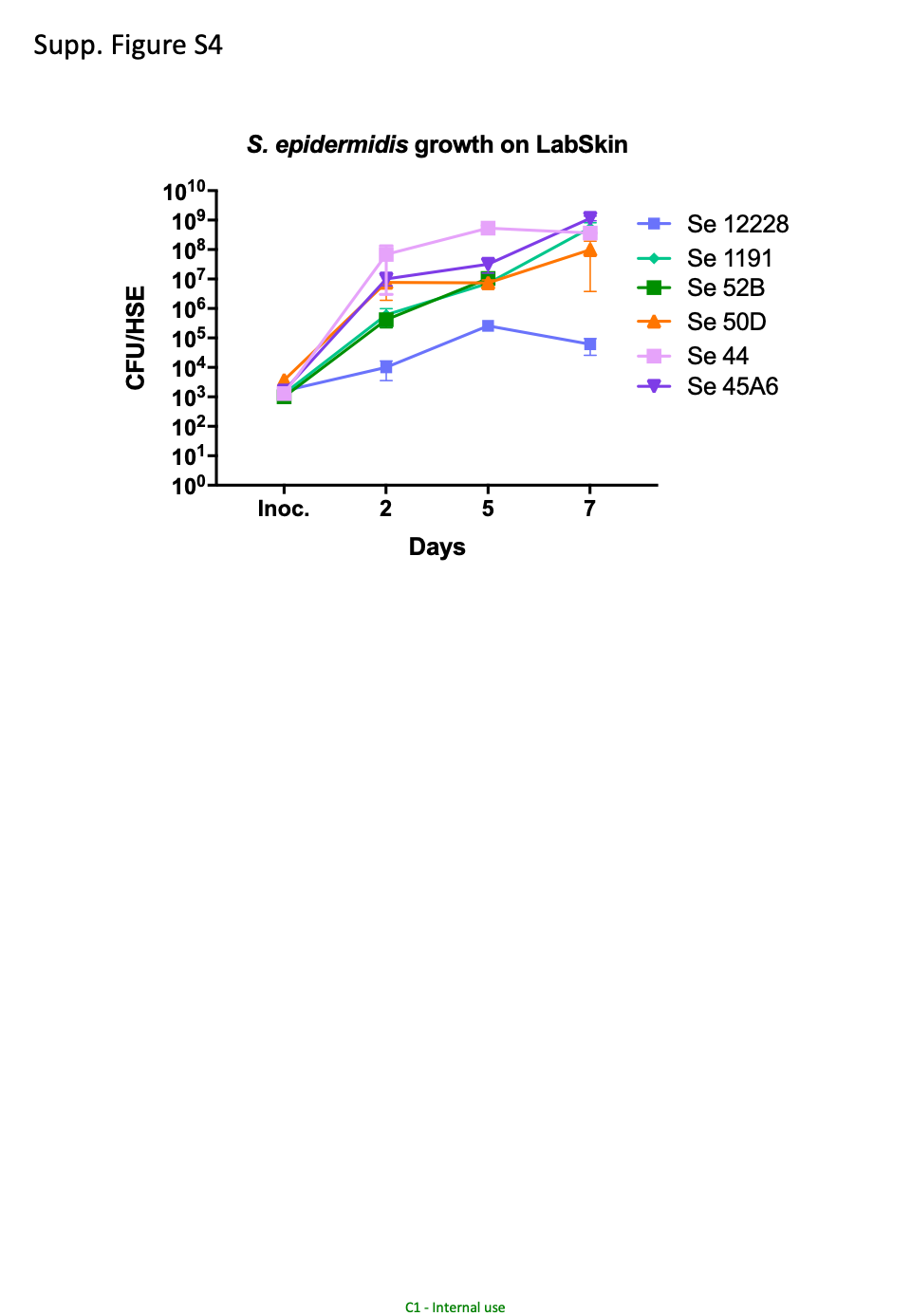

Supplement: Supplementary Figure 4 — S. epidermidis growth on LabSkin HSE model from D0 (inoculation) to D7 post inoculum. The reference strain ATCC 12228 is in blue, the strains from non hyperseborrheic skin in green (1191, 52B), the strain from hyperseborrheic skin in orange (50D) and the strains from atopic skin in purple (44, 45A6). [file Image_4.tiff]

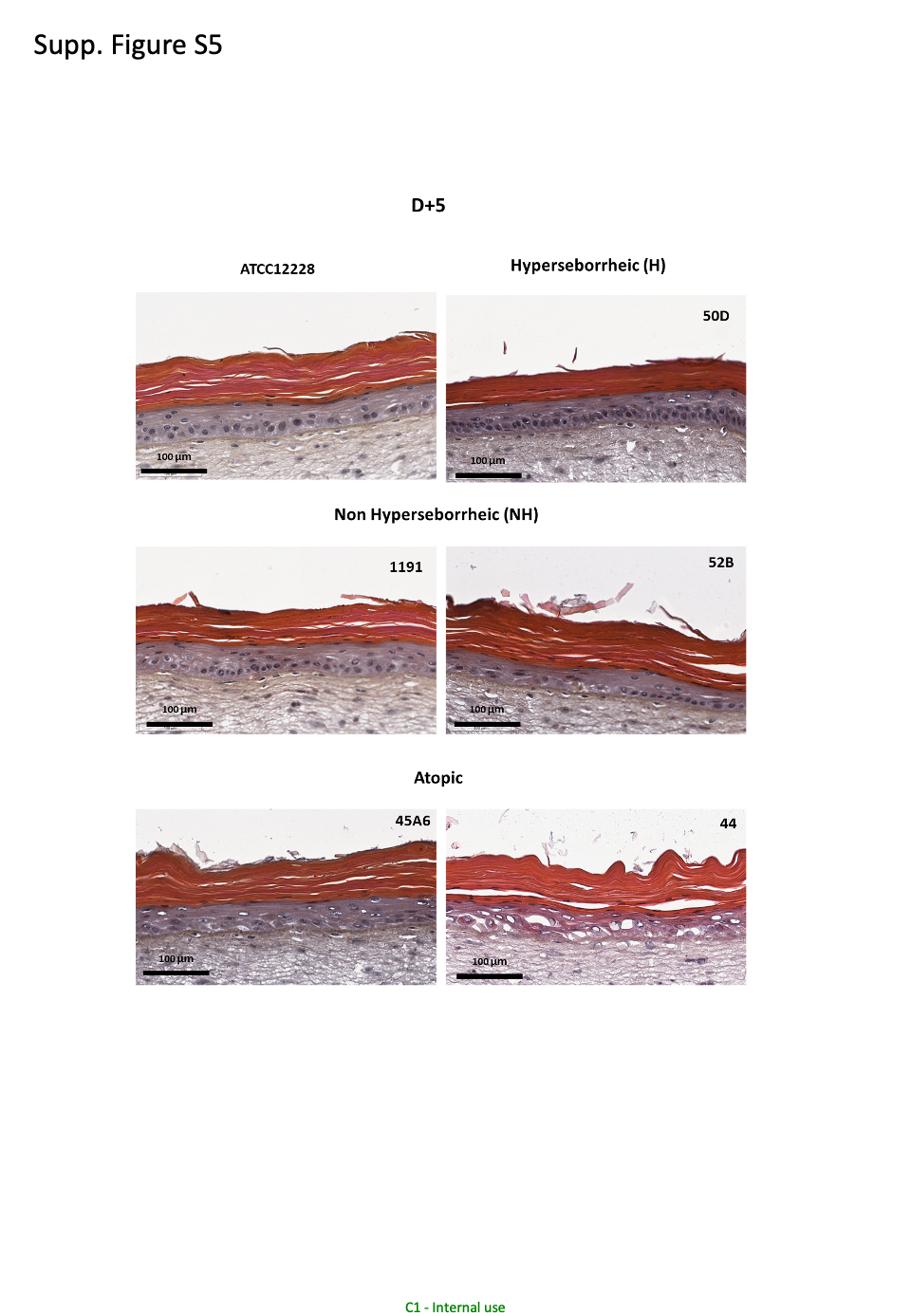

Supplement: Supplementary Figure 5 — Live S. epidermidis effect on keratinocytes depends on skin type origin. Magnification part of HES images of tissues colonized with representative strains of the three skin types origins. [file Image_5.tiff]

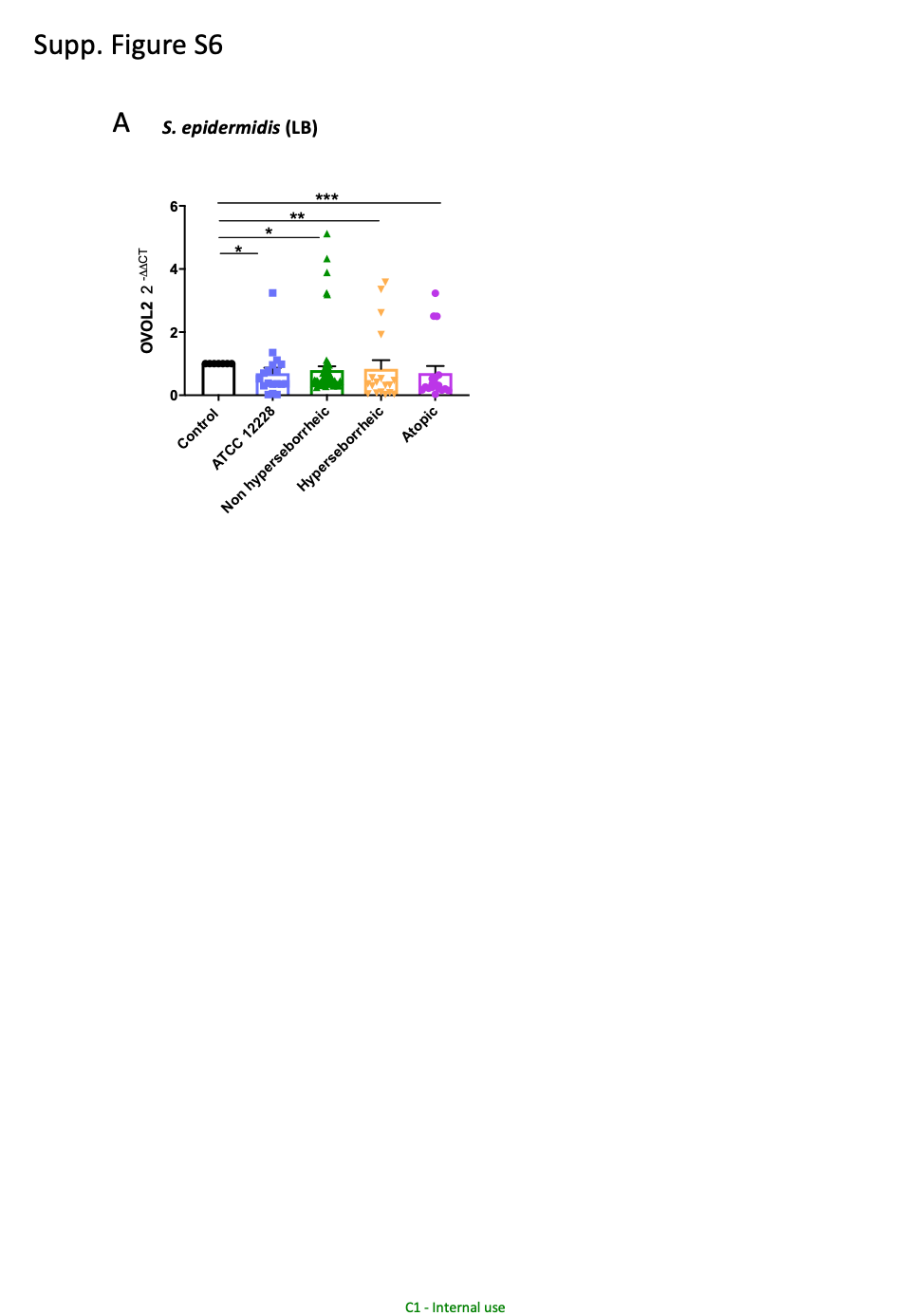

Supplement: Supplementary Figure 6 — Average, per group of skin type, of OVOL2 mRNA expression in NHEK co-cultivated with one of the 11 S. epidermidis isolates or the reference strain ATCC 12228. [file Image_6.tiff]

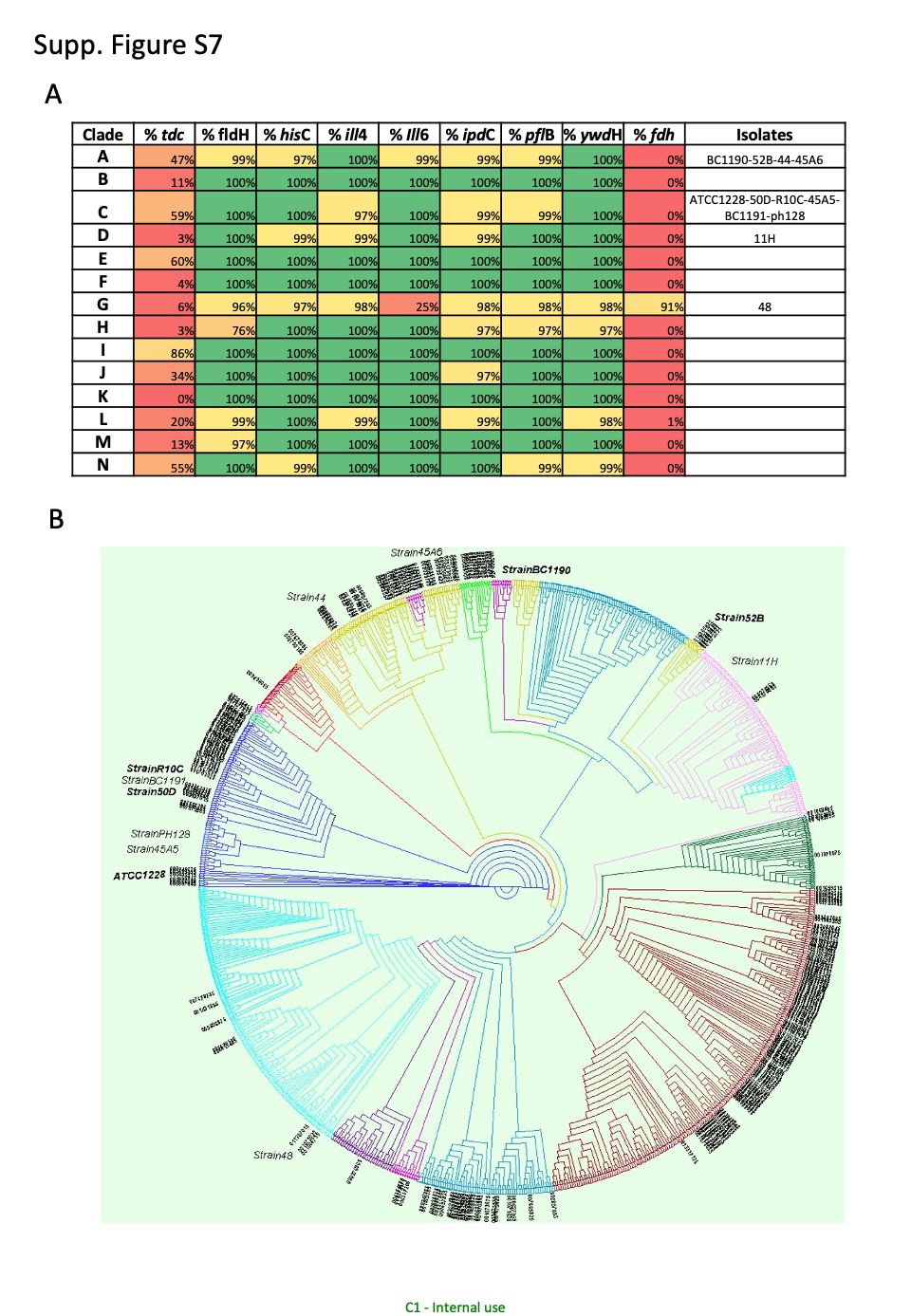

Supplement: Supplementary Figure 7 — (A): Heatmap of positive isolates in each clade for each gene implicated in indole metabolites biosynthesis pathway; (B) cladogram to localize each of the 11 isolates within the previous described clades. [file Image_7.tiff]
